# Synthesis and Evaluation of Ester Derivatives of 10-Hydroxycanthin-6-one as Potential Antimicrobial Agents

**DOI:** 10.3390/molecules21030390

**Published:** 2016-03-21

**Authors:** Fei Zhao, Jiang-Kun Dai, Dan Liu, Shi-Jun Wang, Jun-Ru Wang

**Affiliations:** College of Science, Northwest A & F University, Yangling 712100, China; zhaofeicaiwei@163.com (F.Z.); daijkun@hotmail.com (J.-K.D.); ld127222@126.com (D.L.); 444795415@163.com (S.-J.W.)

**Keywords:** 10-hydroxycanthin-6-one, ester derivatives, antibacterial activity, antifungal activity

## Abstract

As part of our continuing research on canthin-6-one antimicrobial agents, a new series of ester derivatives of 10-hydroxycanthin-6-one were synthesized using a simple and effective synthetic route. The structure of each compound was characterized by NMR, ESI-MS, FT-IR, UV, and elemental analysis. The antimicrobial activity of these compounds against three phytopathogenic fungi (*Alternaria solani*, *Fusarium graminearum*, and *Fusarium solani*) and four bacteria (*Bacillus cereus*, *Bacillus subtilis*, *Ralstonia solanacearum*, and *Pseudomonas syringae*) were evaluated using the mycelium linear growth rate method and micro-broth dilution method, respectively. The structure-activity relationship is discussed. Of the tested compounds, **4** and **7s** displayed significant antifungal activity against *F. graminearum*, with inhibition rates of 100% at a concentration of 50 μg/mL. Compounds **5**, **7s**, and **7t** showed the best inhibitory activity against all the tested bacteria, with minimum inhibitory concentrations (MICs) between 3.91 and 31.25 μg/mL. Thus, **7s** emerged as a promising lead compound for the development of novel canthine-6-one antimicrobial agents.

## 1. Introduction

**Figure 1:**
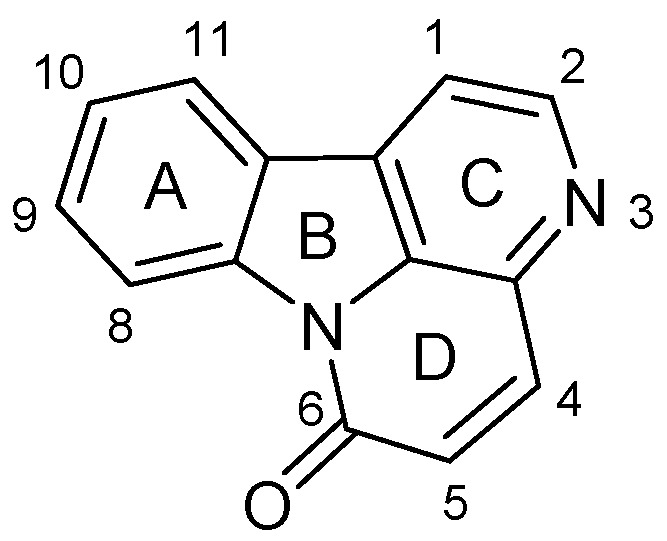
Structure of canthin-6-one.

The canthine-6-one alkaloids represent a fairly large subclass of β-carboline. The first member (Figure 1) in the series was isolated in 1952 from *Pentaceras australis* [1]. Since then, more than 60 compounds with the characteristic tetracyclic core have been isolated from many other species, principally from the Rutaceae and Simaroubaceae families, and recently from fungi [2]. These compounds exhibited a broad spectrum of biological properties, including antiviral [3,4,5], anticancer [6,7,8], antiparasitic [9,10,11], anti-inflammatory [12,13], antibacterial [14,15,16], and antifungal [17,18] activity. Notably, canthine-6-one alkaloids have received considerable attention owing to their high antimicrobial potencies. Soriano-Agaton *et al.* [17] reported the antifungal activity of four naturally occurring canthin-6-ones and their derivatives against five pathogenic fungi. However, the structure-activity relationship (SAR) for the antifungal activity remained unclear. Lagoutte *et al.* [18] investigated the mechanism of action of the antifungal canthin-6-one series in *Saccharomyces cerevisiae*. They showed an affinity of canthine-6-ones for lipid droplets and an effect on lipid metabolism suggesting a stimulation of alkyl chain desaturase enzyme systems. In 2007, O’Donnell *et al.* [16] isolated two canthin-6-ones which displayed minimum inhibitory concentrations (MICs) in the range 8–32 µg/mL against a panel of fast-growing *Mycobacterium* species and 8–64 µg/mL against multidrug-resistant and methicillin-resistant strains of *Staphylococcus aureus*. In a program screening of antimicrobials from natural products and their derivatives, our group [14,19] synthesized a series of hydrophilic derivatives of canthin-6-one having a peak MIC of 0.98 μg/mL against *S. aureus*.

Plant diseases caused by microorganisms have been recognized as a worldwide threat to crop production [20], and they are extremely difficult to manage in agricultural production. Moreover, the repeated use of conventional antimicrobials over many years has resulted in serious environmental and resistance problems. Libraries of natural products are expected to provide a rich resource for the discovery of new drugs [21]. In the past decades, natural product-based or -derived plant protectants have attracted lots of attention due to their low toxicity and good biocompatibility [22].

To exploit the potential antimicrobial activities of compounds having a canthin-6-one skeleton, a novel series of ester derivatives of 10-hydroxycanthin-6-one were synthesized and evaluated for their bioactivity against three phytopathogenic fungi and four bacterial strains. Furthermore, the structure-activity relationship was discussed.

## 2. Results and Discussion

### 2.1. Chemistry

**Scheme 1:**
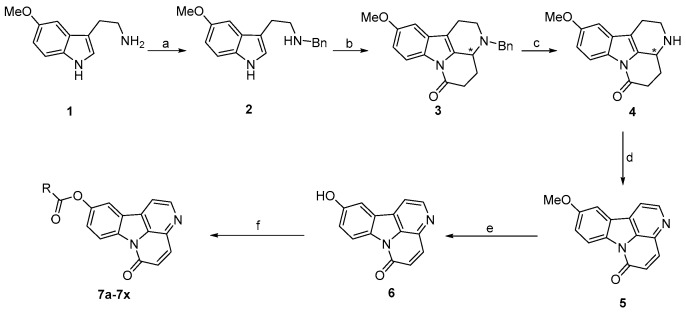
Synthetic route and chemical structures of compounds **4**–**6** and **7a**–**7x**. *Reagents and conditions:* (a) i: benzaldehyde, methanol, r.t.; ii: NaBH_4_, r.t., 98%; (b) *α*-ketoglutaric acid, *p*-TSA, dry toluene: dioxane = 3:2, DST, reflux, 83%; (c) HCOONH_4_, 5% Pd/C, methanol: toluene = 1:1, reflux, 75%; (d) 5% Pd/C, xylene, reflux, 90%; (e) BBr_3_, dry DCM, −78 °C to r.t., 63%; (f) acyl chloride, TEA, dry DCM, 0 °C, 48%–86%.

The synthetic route used for the target compounds is outlined in Scheme 1. The natural product 10-methoxycanthin-6-one (**5**) was synthesized using a modification of previously reported methods [23], with 5-methoxytryptamine (**1**) as the starting material. Another key parent compound, 10-hydroxycanthin-6-one (**6**), was obtained by treating compound **5** with boron tribromide under an argon atmosphere, with anhydrous dichloromethane as solvent. At 0 °C, a new series of ester derivatives of 10-hydroxycanthin-6-one (**7a**–**7x**) was obtained in 48%–86% yield by the reaction of compound **6** with the corresponding acyl chloride in the presence of triethylamine.

The structures of all compounds were elucidated by spectroscopic analysis, including ESI-MS, ^1^H-NMR spectra, ^13^C-NMR spectra, FT-IR, UV, and elemental analysis. All the spectral and analytical data obtained were in agreement with the assigned structures.

### 2.2. Biological Evaluation

#### 2.2.1. Antifungal Activity

The *in vitro* antifungal properties of synthesized compounds **4**–**6** and **7a**–**7x** were evaluated against three phytopathogenic fungi (*Alternaria solani*, *Fusarium graminearum*, and *Fusarium solani*) using the mycelium linear growth rate method [24]. Thiabendazole (TBZ), a commercially available agricultural fungicide, was used as a positive control. The results are summarized in Table 1.

As shown in Table 1, all the compounds displayed activity to some degree against each of the tested fungi. In general, the compounds showed the highest activity against *F. graminearum*, followed by *F. solani*. At a concentration of 50 μg/mL, 58% of the test items (47/81) revealed good activity, with inhibition rates of 49.9%–100%. Compounds **4** and **7s** had the highest activity against *F. graminearum*, with inhibition rates of 100%, equal to that of TBZ. Two compounds (**5** and **7x**) had inhibition rates exceeding 70% against *A. solani.* Six compounds (**4**, **7n**, **7p**, **7s**, **7v**, and **7x**) displayed more than 80% inhibition activity against *F. graminearum*, and four compounds (**5**, **7g**, **7s**, and **7t**) had over 80% inhibition activity against *F. solani*. Of these compounds, compounds **5**, **7s**, and **7t** showed broad-spectrum fungicidal activity against all the phytopathogenic fungi tested. Compound **7g** showed special activity against *F. solani*, displaying 86.1% inhibition activity, but had a low level of activity against *F. graminearum* and *A. solani*. Compound **7p** exhibited much higher activity against *F. graminearum* than against *F. solani* and *A. solani*.

#### 2.2.2. Antibacterial Activity

The antibacterial activity of the compounds was investigated using a previously reported method [14,25]. First, compounds **4**–**6** and **7a**–**7x** (10 μg) were screened for their antibacterial activity against two Gram-positive (*Bacillus cereus* and *Bacillus subtilis*) and two Gram-negative bacterial strains (*Ralstonia solanacearum* and *Pseudomonas syringae*) using the disk diffusion method. Penicillin sodium was used as a positive control, and a dichloromethane poured disk as a negative control. The diameter of the inhibition zone was assayed to make a preliminary evaluation of the antibacterial potential of the tested compounds. The results are summarized in Table 2.

The results revealed that compounds **4**, **7a**–**7r**, and **7x** showed no antibacterial activity (growth inhibition zones < 8 mm), while compounds **5**, **6**, and **7s**–**7w** exhibited moderate activity against both Gram-positive and Gram-negative bacteria. Compounds **7s** and **7t** showed higher antibacterial activities than the parent compounds **5** and **6**. However, compounds **7u**–**7w** exhibited equivalent or weaker antibacterial activity than compound **5**.

The MICs of compounds **5**, **6**, and **7s**–**7w** were determined in further studies using the micro-broth dilution method. The results are shown in Table 3.

Significant activity was observed for compounds **5**, **7s**, and **7t** against all the tested bacterial strains, with MICs between 3.91 and 31.25 μg/mL. Compound **5** was active against the tested Gram-positive bacteria, with MICs equivalent to that of penicillin sodium. However, none of the investigated derivatives were found to be superior to the reference drug.

### 2.3. Structure-Activity Relationship

**Figure 2:**
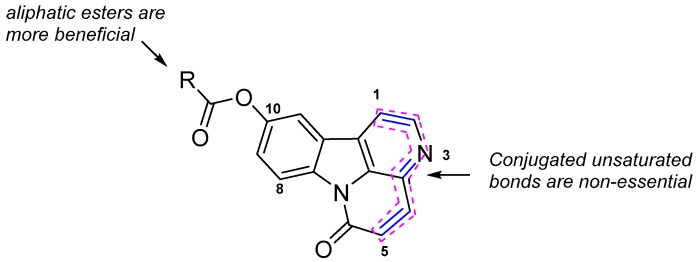
Structure-activity relationship for the antifungal activity.

Based on the results of the present study, the structure–activity relationship (SAR) for the antifungal activity may be summarized as shown in Figure 2. Comparison of the antifungal activities of compound **4** and 10-methoxycanthin-6-one (**5**) suggests that the conjugated unsaturated bonds of rings C and D (see Figure 1) are non-essential. The aliphatic ester derivatives of 10-hydroxycanthin-6-one had much higher antifungal activity than did the aromatic ester derivatives.

**Figure 3:**
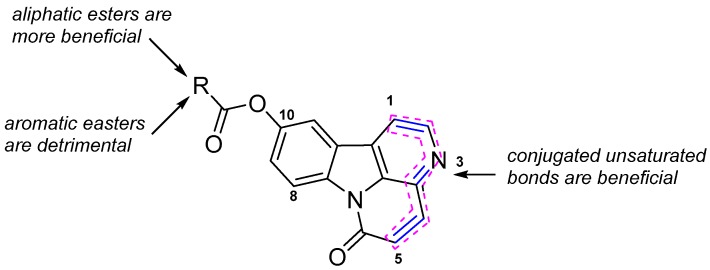
Structure-activity relationship for the antibacterial activity.

The weak antibacterial activity of compound **4** suggests that the conjugated unsaturated bonds of rings C and D play an important role in antibacterial activity. The introduction of aromatic esters decreases the antibacterial activity. Other compounds with a shorter or longer aliphatic chain did not show any distinct effect, except for compound **7x** (Figure 3).

## 3. Materials and Methods

### 3.1. General Details

All the reagents and solvents were obtained locally and were of analytical grade or were purified according to standard methods before use. The tested fungi and bacteria (*B. cereus*, *B. subtilis*, *R. solanacearum*, *P. syringae*, *A. solani*, *F. graminearum*, and *F. solani*) were provided by the Institute of Pesticide Science, Northwest A&F University, China. Melting points were determined using a digital melting-point apparatus and were uncorrected. ^1^H-NMR (500 MHz) and ^13^C-NMR (125 MHz) spectra were recorded using a Bruker Avance III 500 MHz instrument (Bruker, Madison, WI, USA), with TMS as the internal standard and CDCl_3_ or dimethylsulfoxide (DMSO-*d*_6_) as the solvent. Chemical shifts (δ values) and coupling constants (*J* values) are given in this paper in ppm and Hz, respectively. ESI-MS was carried out using a Thermo Fisher LCQ Fleet instrument (Shimadzu, Beijing, China). Elemental analysis of the synthesized compounds was undertaken using a CE-440 elemental analyzer (EAI, North Chelmsford, MA, USA). Ultraviolet spectra (UV) were obtained using a Thermo Scientific Evolution 300 UV-vis spectrophotometer (Shimadzu, Beijing, China). Infrared spectra (IR) were recorded on a Bruker TENSOR 27 spectrometer (Bruker, Madison, WI, USA) with KBr disks.

### 3.2. Synthesis of Target Compounds

#### 3.2.1. Synthesis of Compound **2**

A previously described method [26] was used to synthesize compound **2**. A solution of 5-methoxytryptamine (**1**) (4.76 g, 25 mmol) and benzaldehyde (3.06 mL, 30 mmol) in methanol was vigorously stirred at room temperature for 36 h. Solid NaBH_4_ (2.84 g, 75 mmol) was added to the resulting solution, and the mixture stirred at room temperature for 1 h. The reaction was quenched by adding saturated aqueous NaHCO_3_ (100 mL), and the mixture concentrated under vacuum until approximately 50 mL remained. The residue was filtered, and the filter cake was washed with CH_2_Cl_2_. The organic phase was separated, and the aqueous phase was extracted with CH_2_Cl_2_ (3 × 50 mL). The combined organic phases were washed with brine (3 × 50 mL) and dried over anhydrous Na_2_SO_4_. The organic solvent was removed under vacuum to give the title compound as a brown solid (6.87 g, yield 98%, m.p. 71.2–72.3 °C), which was used without further purification. ^1^H-NMR (500 MHz, CDCl_3_) δ: 8.06 (s, 1H), 7.37–7.28 (m, 2H), 7.26–7.18 (m, 4H), 7.02 (d, *J* = 2.5 Hz, 1H), 6.95 (d, *J* = 2.2 Hz, 1H), 6.84 (dd, *J* = 8.8, 2.4 Hz, 1H), 3.87–3.76 (m, 5H), 3.01–2.93 (m, 4H), 1.87(s, 1H). ^13^C-NMR (125 MHz, CDCl_3_) δ: 153.9, 140.1, 131.6, 128.4, 128.2, 127.9, 127.0, 122.8, 113.5, 112.3, 111.9, 100.8, 56.0, 53.9, 49.3, 25.8. ESI-MS *m*/*z*: [M + H]^+^_(found)_ = 281.16, [M + H]^+^_(calcd)_ = 281.37. Elemental analysis calculated for C_18_H_20_N_2_O (%): C, 77.11; H, 7.19; N, 9.99 (found: C, 77.02; H, 7.23; N, 10.05). ^1^H-NMR, ^13^C-NMR, and FT-IR spectra for some target compounds were showed in the Supplementary Materials.

#### 3.2.2. Synthesis of Compound **3**

A mixture of compound **2** (5.61 g, 20 mmol), *α*-ketoglutaric acid (3.51 g, 24 mmol), *p*-toluenesulfonic acid (0.69 g, 4 mmol), and a solution of toluene/dioxane (60 mL 3:2 *v*/*v*) was heated at 180 °C for 48 h, using a Dean-Stark trap for water removal. Upon completion, the reaction mixture was cooled to room temperature and the organic solvent removed. The residue was dissolved in a mixture of CH_2_Cl_2_ (60 mL) and saturated aqueous NaHCO_3_ (80 mL). The aqueous phase was separated and the product extracted with CH_2_Cl_2_ (3 × 50 mL). The combined organic phases were washed with brine (3 × 50 mL) and dried over anhydrous Na_2_SO_4_. The obtained crude product was chromatographed on silica gel eluting with dichloromethane to afford compound **3** as a white powder (5.75 g, yield 83%, m.p. 192.5–193.7 °C). ^1^H-NMR (500 MHz, CDCl_3_) δ: 8.26 (d, *J* = 8.8 Hz, 1H), 7.43–7.27 (m, 5H), 6.89 (dd, *J* = 8.8, 2.5 Hz, 1H), 6.86 (d, *J* = 2.5 Hz, 1H), 4.23 (d, *J* = 13.5 Hz, 1H), 3.85 (s, 3H), 3.56–3.52 (m, 1H), 3.39 (d, *J* = 13.5 Hz, 1H), 3.25–3.15 (m, 1H), 2.91–2.68 (m, 3H), 2.61–2.47 (m, 3H), 1.96–1.85 (m, 1H). ^13^C-NMR (125 MHz, CDCl_3_) δ: 167.7, 156.9, 138.5, 135.4, 130.5, 129.6, 128.9, 128.5, 127.3, 116.9, 113.4, 112.0, 101.8, 57.7, 57.3, 55.7, 50.1, 32.8, 27.6, 21.4. ESI-MS *m*/*z*: [M + H]^+^_(found)_ = 347.23, [M + H]^+^_(calcd)_ = 347.43. Elemental analysis calculated for C_22_H_22_N_2_O_2_ (%): C, 76.28; H, 6.40; N, 8.09 (found: C, 76.12; H, 6.51; N, 8.03).

#### 3.2.3. Synthesis of Compound **4**

To a flask was added compound **3** (5.75 g, 16.6 mmol), HCOONH_4_ (10.52 g, 167 mmol), Pd/C (5%, 1.5 g), methanol (25 mL), and toluene (25 mL). The mixture was stirred vigorously at 80 °C. After 4 h, the reaction was quenched by addition of saturated aqueous NaHCO_3_ until pH 8.5 was reached. The mixture was filtered, and the filter cake was washed with copious amounts of dichloromethane and methanol until TLC indicated complete removal of the product. The combined filtrates were concentrated under vacuum until approximately 50 mL remained, and then extracted with CH_2_Cl_2_ (3 × 80 mL). The combined organic phases were washed with brine (3 × 80 mL) and dried over anhydrous Na_2_SO_4_. The sample was concentrated and purified using silica gel column chromatography, eluting with ethyl acetate-methanol (*v*/*v* = 2:1) to give product **4** as a green solid (3.19 g, yield 75%, m.p. > 250 °C). ^1^H-NMR (500 MHz, CDCl_3_) δ 8.24 (d, *J* = 8.9, 1H), 6.92–6.85 (m, 2H), 4.02–3.95 (m, 1H), 3.86 (s, 3H), 3.56–3.49 (m, 1H), 3.18–3.10 (m, 1H), 2.86–2.67 (m, 3H), 2.66–2.58 (m, 1H), 2.33–2.24 (m, 1H), 1.85–1.74 (m, 2H). ^13^C-NMR (125 MHz, CDCl_3_) δ: 167.8, 156.9, 136.9, 131.0, 129.2, 116.8, 112.8, 112.0, 101.9, 55.8, 50.7, 43.9, 33.1, 29.2, 21.7. ESI-MS *m*/*z*: [M + H]^+^_(found)_ = 257.09, [M + H]^+^_(calcd)_ = 257.31. Elemental analysis calculated for C_15_H_16_N_2_O_2_ (%): C, 70.29; H, 6.29; N, 10.93 (found: C, 70.33; H, 6.26; N, 10.89).

#### 3.2.4. Synthesis of Compound **5**

To a stirred solution of compound **4** (2.56 g, 10 mmol) in xylene was added Pd/C (5%, 1.8 g), and the mixture was heated at reflux for 4 days. The mixture was then cooled and filtered, and the filtered solid washed carefully with dichloromethane and methanol. The combined filtrates were concentrated under vacuum and purified using column chromatography on silica gel using dichloromethane–acetone (*v*/*v* = 5:1) as eluent to give compound **5** as a bright yellow solid (2.25 g, yield 90%, m.p. 202.9–204.1 °C). IR, υ_max_ (KBr) cm^−1^: 1676 (C=O), 1450 (C=N). UV λ_max_ (lg ε) (DCM) 380 (3.91) nm. ^1^H-NMR (500 MHz, DMSO-*d*_6_) δ: 8.84 (d, *J* = 5.0 Hz, 1H), 8.38 (d, *J* = 8.9 Hz, 1H), 8.30 (d, *J* = 5.0 Hz, 1H), 8.12 (d, *J* = 9.8 Hz, 1H), 7.99 (d, *J* = 2.6 Hz, 1H), 7.34 (dd, *J* = 8.9, 2.6 Hz, 1H), 6.99 (d, *J* = 9.8 Hz, 1H), 3.92 (s, 3H). ^13^C-NMR (125 MHz, CDCl_3_) δ: 159.3, 157.9, 145.7, 139.3, 136.4, 133.8, 132.5, 130.3, 129.0, 125.7, 118.0, 118.0, 116.4, 106.5, 56.0. ESI-MS *m*/*z*: [M + H]^+^_(found)_ = 251.25, [M + H]^+^_(calcd)_ = 251.26. Elemental analysis calculated for C_15_H_10_N_2_O_2_ (%): C, 71.99; H, 4.03; N, 11.19 (found: C, 72.03; H, 4.12; N, 11.07).

#### 3.2.5. Synthesis of Compound **6**

To a solution of 10-methoxycanthin-6-one (2.25 g, 9 mmol) in anhydrous dichloromethane (100 mL) was added dropwise boron tribromide (8.50 mL, 90 mmol) under argon at −78 °C, and the mixture stirred overnight at room temperature. The reaction was cautiously quenched with iced water (80 mL). The solids were filtered and the filter cake washed with dichloromethane and then purified using silica gel column chromatography to give 10-hydroxycanthin-6-one (**6**) as a yellow powder (1.34 g, yield 63%, m.p. > 250 °C). IR, υ_max_ (KBr) cm^−1^: 3424 (OH), 1691 (C=O), 1436 (C=N). UV λ_max_ (lg ε) (DCM) 375 (3.89) nm. ^1^H-NMR (500 MHz, DMSO-*d*_6_) δ: 10.01 (s, 1H), 8.79 (d, *J* = 5.0 Hz, 1H), 8.30 (d, *J* = 8.8 Hz, 1H), 8.23 (d, *J* = 5.0 Hz, 1H), 8.08 (d, *J* = 9.8 Hz, 1H), 7.67 (d, *J* = 2.5 Hz, 1H), 7.19 (dd, *J* = 8.8, 2.5 Hz, 1H), 6.96 (d, *J* = 9.8 Hz, 1H). ^13^C-NMR (125 MHz, DMSO-*d*_6_) δ 158.9, 156.2, 146.1, 139.7, 136.2, 132.4, 132.2, 130.0, 129.0, 126.0, 119.0, 117.7, 117.4, 109.5.ESI-MS *m*/*z*: [M + H]^+^_(found)_ = 237.21, [M + H]^+^_(calcd)_ = 237.23. Elemental analysis calculated for C_14_H_8_N_2_O_2_ (%): C, 71.18; H, 3.41; N, 11.86 (found: C, 71.20; H, 3.45; N, 11.77).

#### 3.2.6. General Procedure for the Synthesis of Compounds **7a**–**7x**

According to the previously reported method [27], compounds **7a**–**7x** were prepared through the direct condensation of 10-hydroxycanthin-6-one (**6**) with the corresponding acyl chloride. The general procedure was as follows. A solution of benzoyl chloride (0.05 mL, 0.45 mmol) in CH_2_Cl_2_ (5 mL) was added slowly to an ice-cooled mixture of 10-hydroxycanthin-6-one (0.71 g, 0.3 mmol) and triethylamine (0.21 mL, 1.5 mmol) in anhydrous CH_2_Cl_2_ (30 mL) over 15 min with stirring. The reaction mixture was stirred overnight at room temperature to provide a clear solution, and then quenched with saturated aqueous NaHCO_3_ (50 mL). The organic phase was separated, washed with brine (3 × 50 mL), and finally dried over anhydrous Na_2_SO_4_. The solvent was removed and the residue purified by column chromatography on silica gel, eluting with dichloromethane–acetone (*v*/*v* = 20:1) to afford **7a** as a pale yellow powder (0.82 g, yield 80%, m.p. 220.4–221.2 °C). IR, υ_max_ (KBr) cm^−1^: 1685 (C=O), 1669 (ester), 1420 (C=N). UV λ_max_ (lg ε) (DCM) 364 (4.25) nm, 383 (4.24) nm. ^1^H-NMR (500 MHz, CDCl_3_) δ 8.87 (d, *J* = 5.0 Hz, 1H), 8.75 (d, *J* = 8.7 Hz, 1H), 8.33–8.25 (m, 2H), 8.09 (d, *J* = 9.8 Hz, 1H), 8.05 (d, *J* = 2.3 Hz, 1H), 7.97 (d, *J* = 5.0 Hz, 1H), 7.76–7.69 (m, 1H), 7.63–7.56 (m, 3H), 7.04 (d, *J* = 9.8 Hz, 1H). ^13^C-NMR (125 MHz, CDCl_3_) δ165.3, 159.3, 148.7, 145.9, 139.6, 137.0, 136.4, 134.0, 130.3, 130.0, 129.8, 129.1, 129.0, 128.7, 125.5, 124.5, 118.0, 116.7, 116.2. ESI-MS *m*/*z*: [M + H]^+^_(found)_ = 341.35, [M + H]^+^_(calcd)_ = 341.34; Elemental analysis calculated for C_21_H_12_N_2_O_3_ (%): C, 74.11; H, 3.55; N, 8.23 (found: C, 74.09; H, 3.45; N, 8.26).

*6-Oxo-6H-Indolo [3,2,1-de] [1,5] naphthyridin-10-yl nicotinate* (**7b**): Yield: 64%; yellow solid; m.p. 235.8–236.8 °C; IR, υ_max_ (KBr) cm^−1^: 1739 (C=O), 1683 (ester), 1588 (C=N). UV λ_max_ (lg ε) (DCM) 364 (4.04) nm, 382 (4.03) nm. ^1^H-NMR (500 MHz, CDCl_3_) δ 9.45 (d, *J* = 2.2 Hz, 1H), 8.90 (dd, *J* = 4.9, 1.7 Hz, 1H), 8.84 (d, *J* = 5.0 Hz, 1H), 8.73 (d, *J* = 8.8 Hz, 1H), 8.50 (dt, *J* = 7.9, 2.0 Hz, 1H), 8.06–8.00 (m, 2H), 7.93 (d, *J* = 5.0 Hz, 1H), 7.56 (dd, *J* = 8.8, 2.3 Hz, 1H), 7.51 (ddd, *J* = 8.0, 4.9, 0.9 Hz, 1H), 6.99 (d, *J* = 9.8 Hz, 1H). ^13^C-NMR (125 MHz, CDCl_3_) δ 164.0, 159.3, 154.3, 151.5, 148.2, 146.0, 139.8, 137.7, 137.2, 136. 6, 132.6, 129.6, 129.0, 125.6, 125.3, 124.3, 123.6, 118.1, 116.6, 116.1. ESI-MS *m*/*z*: [M + H]^+^_(found)_ = 342.34, [M + H]^+^_(calcd)_ = 342.33; Elemental analysis calculated for C_20_H_11_N_3_O_3_ (%): C, 70.38; H, 3.25; N, 12.31 (found: C, 70.25; H, 3.13; N, 12.28).

*6-Oxo-6H-Indolo [3,2,1-de] [1,5] naphthyridin-10-yl 2-methylbenzoate* (**7c**): Yield: 85%; yellow powder; m.p. 214.7–215.8 °C; IR, υ_max_ (KBr) cm^−1^: 1715 (C=O), 1667 (ester), 1423 (C=N). UV λ_max_ (lg ε) (DCM) 364 (4.26) nm, 382 (4.24) nm. ^1^H-NMR (500 MHz, CDCl_3_) δ 8.84 (d, *J* = 4.9 Hz, 1H), 8.72 (d, *J* = 8.8 Hz, 1H), 8.23 (d, *J* = 7.9 Hz, 1H), 8.04 (d, *J* = 9.8 Hz, 1H), 8.01 (d, *J* = 2.2 Hz, 1H), 7.94 (d, *J* = 5.0 Hz, 1H), 7.57–7.49 (m, 2H), 7.40–7.33 (m, 2H), 7.00 (d, *J* = 9.8 Hz, 1H), 2.72 (s, 3H). ^13^C-NMR (125 MHz, CDCl_3_) δ 165.8, 159.4, 148.7, 146.0, 141.6, 139.7, 137.0, 136.5, 133.1, 132.6, 132.1, 131.3, 129.8, 129.0, 128.1, 126.1, 125.6, 124.6, 118.0, 116.6, 116.3, 22.0. ESI-MS *m*/*z*: [M + H]^+^_(found)_ = 355.33, [M + H]^+^_(calcd)_ = 355.37; Elemental analysis calculated for C_22_H_14_N_2_O_3_ (%): C, 74.57; H, 3.98; N, 7.91 (found: C, 74.62; H, 4.03; N, 7.88).

*6-Oxo-6H-Indolo [3,2,1-de] [1,5] naphthyridin-10-yl 3-methylbenzoate* (**7d**): Yield: 80%; pale yellow powder; m.p. 183.2–183.9 °C; IR, υ_max_ (KBr) cm^−1^: 1738 (C=O), 1636 (ester), 1420 (C=N). UV λ_max_ (lg ε) (DCM) 364 (4.26) nm, 382 (4.24) nm. ^1^H-NMR (500 MHz, CDCl_3_) δ 8.81 (dd, *J* = 6.6, 4.8 Hz, 1H), 8.69 (t, *J* = 8.9 Hz, 1H), 8.07–7.97 (m, 4H), 7.90 (dd, *J* = 8.9, 4.9 Hz, 1H), 7.54 (dd, *J* = 8.8, 2.3 Hz, 1H), 7.48 (d, *J* = 7.7 Hz, 1H), 7.42 (td, *J* = 7.5, 2.9 Hz, 1H), 6.98 (dd, *J* = 9.8, 5.9 Hz, 1H), 2.46 (d, *J* = 2.3 Hz, 3H). ^13^C NMR (125 MHz, CDCl_3_) δ 165.4, 159.3, 148.7, 145.9, 139.6, 138.6, 137.0, 136.5, 134.7, 132.5, 130.8, 129.7, 129.1, 129.0, 128.6, 127.4, 125.5, 124.5, 118.0, 116.6, 116.2, 21.3. ESI-MS *m*/*z*: [M + H]^+^_(found)_ = 355.35, [M + H]^+^_(calcd)_ = 355.37; Elemental analysis calculated for C_22_H_14_N_2_O_3_ (%): C, 74.57; H, 3.98; N, 7.91 (found: C, 74.62; H, 3.96; N, 7.97).

*6-Oxo-6H-Indolo [3,2,1-de] [1,5] naphthyridin-10-yl 4-methylbenzoate* (**7e**): Yield: 83%; pale yellow powder; m.p. 227.9–228.5 °C; IR, υ_max_ (KBr) cm^−1^: 1732 (C=O), 1636 (ester), 1421 (C=N). UV λ_max_ (lg ε) (DCM) 364 (4.22) nm, 382 (4.20) nm. ^1^H-NMR (500 MHz, CDCl_3_) δ 8.83 (d, *J* = 5.0 Hz, 1H), 8.71 (d, *J* = 8.8 Hz, 1H), 8.14 (d, *J* = 8.1 Hz, 2H), 8.03 (d, *J* = 9.8 Hz, 1H), 8.01 (d, *J* = 2.2 Hz, 1H), 7.93 (d, *J* = 5.0 Hz, 1H), 7.55 (dd, *J* = 8.8, 2.3 Hz, 1H), 7.35 (d, *J* = 8.0 Hz, 2H), 7.00 (d, *J* = 9.8 Hz, 1H), 2.48 (s, 3H). ^13^C-NMR (125 MHz, CDCl_3_) δ 165.3, 159.4, 148.8, 146.0, 144.9, 139.7, 137.0, 136.5, 132.6, 130.3, 129.8, 129.5, 129.0, 126.4, 125.5, 124.6, 118.0, 116.6, 116.3, 21.8. ESI-MS *m*/*z*: [M + H]^+^_(found)_ = 355.35, [M + H]^+^_(calcd)_ = 355.37; Elemental analysis calculated for C_22_H_14_N_2_O_3_ (%): C, 74.57; H, 3.98; N, 7.91 (found: C, 74.43; H, 3.99; N, 7.86).

*6-Oxo-6H-Indolo [3,2,1-de] [1,5] naphthyridin-10-yl 2-chlorobenzoate* (**7f**): Yield: 72%; pale yellow powder; m.p. 208.9–210.3 °C; IR, υ_max_ (KBr) cm^−1^: 1744 (C=O), 1676 (ester), 1416 (C=N). UV λ_max_ (lg ε) (DCM) 364 (4.31) nm, 382 (4.32) nm. ^1^H-NMR (500 MHz, CDCl_3_) δ 8.84 (d, *J* = 5.0 Hz, 1H), 8.72 (d, *J* = 8.8 Hz, 1H), 8.11 (dd, *J* = 7.8, 1.2 Hz, 1H), 8.06–8.02 (m, 2H), 7.94 (d, *J* = 5.0 Hz, 1H), 7.59–7.52 (m, 3H), 7.46–7.41 (m, 1H), 6.99 (d, *J* = 9.8 Hz, 1H). ^13^C-NMR (125 MHz, CDCl_3_) δ 164.1, 159.4, 148.3, 146.0, 139.7, 137.1, 136.5, 134.6, 133.6, 132.6, 132.0, 131.5, 129.7, 129.0, 128.9, 126.9, 125.6, 124.4, 118.1, 116.7, 116.1. ESI-MS *m*/*z*: [M + H]^+^_(found)_ = 375.33, [M + H]^+^_(calcd)_ = 375.78; Elemental analysis calculated for C_21_H_11_ClN_2_O_3_ (%): C, 67.30; H, 2.96; N, 7.47 (found: C, 67.22; H, 2.98; N, 7.53).

*6-Oxo-6H-Indolo [3,2,1-de] [1,5] naphthyridin-10-yl 3-chlorobenzoate* (**7g**): Yield: 75%; pale yellow powder; m.p. 240.2–241.1 °C; IR, υ_max_ (KBr) cm^−1^: 1742 (C=O), 1677 (ester), 1422 (C=N). UV λ_max_ (lg ε) (DCM) 364 (4.33) nm, 382 (4.34) nm. ^1^H-NMR (500 MHz, CDCl_3_) δ 8.85 (d, *J* = 5.0 Hz, 1H), 8.75 (d, *J* = 8.8 Hz, 1H), 8.24 (t, *J* = 1.8 Hz, 1H), 8.14 (dt, *J* = 7.8, 1.2 Hz, 1H), 8.05 (d, *J* = 9.8 Hz, 1H), 8.02 (d, *J* = 2.2 Hz, 1H), 7.95 (d, *J* = 5.0 Hz, 1H), 7.69–7.63 (m, 1H), 7.56 (dd, *J* = 8.8, 2.3 Hz, 1H), 7.51 (t, *J* = 7.9 Hz, 1H), 7.01 (d, *J* = 9.8 Hz, 1H). ^13^C-NMR (125 MHz, CDCl_3_) δ 164.1, 159.4, 148.4, 146.0, 139.8, 137.2, 136.6, 135.0, 134.0, 132.6, 131.0, 130.3, 130.1, 129.7, 129.0, 128.4, 125.6, 124.4, 118.1, 116.6, 116.1. ESI-MS *m*/*z*: [M + H]^+^_(found)_ = 375.33, [M + H]^+^_(calcd)_ = 375.78; Elemental analysis calculated for C_21_H_11_ClN_2_O_3_ (%): C, 67.30; H, 2.96; N, 7.47 (found: C, 67.33; H, 3.04; N, 7.32).

*6-Oxo-6H-Indolo [3,2,1-de] [1,5] naphthyridin-10-yl 4-chlorobenzoate* (**7h**): Yield: 69%; pale yellow powder; m.p. 239.8–240.7 °C; IR, υ_max_ (KBr) cm^−1^: 1734 (C=O), 1684 (ester), 1420 (C=N). UV λ_max_ (lg ε) (DCM) 364 (4.53) nm, 382(4.52) nm. ^1^H-NMR (500 MHz, CDCl_3_) δ 8.82 (d, *J* = 5.0 Hz, 1H), 8.71 (d, *J* = 8.8 Hz, 1H), 8.18 (d, *J* = 8.6 Hz, 2H), 8.03 (d, *J* = 9.8 Hz, 1H), 7.99 (d, *J* = 2.2 Hz, 1H), 7.91 (d, *J* = 5.0 Hz, 1H), 7.57–7.49 (m, 3H), 6.99 (d, *J* = 9.8 Hz, 1H). ^13^C-NMR (125 MHz, CDCl_3_) δ 164.4, 159.3, 148.4, 146.0, 140.6, 139.7, 137.1, 136.5, 132.6, 131.6, 129.7, 129.1, 129.0, 127.6, 125.6, 124.4, 118.0, 116.6, 116.1. ESI-MS *m*/*z*: [M + H]^+^_(found)_ = 375.35, [M + H]^+^_(calcd)_ = 375.78; Elemental analysis calculated for C_21_H_11_ClN_2_O_3_ (%): C, 67.30; H, 2.96; N, 7.47 (found: C, 67.36; H, 2.95; N, 7.53).

*6-Oxo-6H-Indolo [3,2,1-de] [1,5] naphthyridin-10-yl 2, 4-dichlorobenzoate* (**7i**): Yield: 78%; pale yellow powder; m.p. 246.2–247.1 °C; IR, υ_max_ (KBr) cm^−1^: 1738 (C=O), 1676 (ester), 1423 (C=N). UV λ_max_ (lg ε) (DCM) 364 (4.23) nm, 383 (4.25) nm. ^1^H-NMR (500 MHz, CDCl_3_) δ 8.84 (d, *J* = 5.0 Hz, 1H), 8.73 (d, *J* = 8.8 Hz, 1H), 8.09 (d, *J* = 8.5, 1H), 8.07–8.01 (m, 2H), 7.95 (d, *J* = 5.0 Hz, 1H), 7.59 (d, *J* = 2.0 Hz, 1H), 7.56 (dd, *J* = 8.8, 2.3 Hz, 1H), 7.43 (dd, *J* = 8.4, 2.0 Hz, 1H), 7.00 (d, *J* = 9.8 Hz, 1H). ^13^C-NMR (125 MHz, CDCl_3_) δ 163.2, 159.4, 148.2, 146.0, 139.8, 139.6, 137.2, 136.6, 135.9, 133.1, 132.6, 131.5, 129.7, 129.0, 127.4, 127.0, 125.6, 124.3, 118.1, 116.7, 116.1. ESI-MS m/z: [M + H]^+^_(found)_ = 410.34, [M + H]^+^_(calcd)_ = 410.22; Elemental analysis calculated for C_21_H_10_Cl_2_N_2_O_3_ (%): C, 61.64; H, 2.46; N, 6.85 (found: C, 61.61; H, 2.38; N, 6.77).

*6-Oxo-6H-Indolo [3,2,1-de] [1,5] naphthyridin-10-yl 4-methoxybenzoate* (**7j**): Yield: 72%; yellow powder; m.p. > 250 °C; IR, υ_max_ (KBr) cm^−1^: 1726 (C=O), 1673 (ester), 1419 (C=N). UV λ_max_ (lg ε) (DCM) 364 (3.98) nm, 382 (3.96) nm. ^1^H-NMR (500 MHz, CDCl_3_) δ 8.84 (d, *J* = 5.0 Hz, 1H), 8.73 (d, *J* = 8.8 Hz, 1H), 8.24–8.18 (m, 2H), 8.05 (d, *J* = 9.8 Hz, 1H), 8.02 (d, *J* = 2.2 Hz, 1H), 7.94 (d, *J* = 5.0 Hz, 1H), 7.55 (dd, *J* = 8.8, 2.3 Hz, 1H), 7.05–6.98 (m, 3H), 3.92 (s, 3H). ^13^C-NMR (125 MHz, CDCl_3_) δ 165.0, 164.2, 159.4, 148.8, 146.0, 139.7, 137.0, 136.5, 132.6, 132.4, 129.9, 129.0, 129.5, 124.6, 121.4, 118.0, 116.6, 116.3, 114.0, 55.6. ESI-MS *m*/*z*: [M + H]^+^_(found)_ = 371.34, [M + H]^+^_(calcd)_ = 371.36; Elemental analysis calculated for C_22_H_14_N_2_O_4_ (%): C, 71.35; H, 3.81; N, 7.56 (found: C, 71.29; H, 3.92; N, 7.49).

*6-Oxo-6H-Indolo [3,2,1-de] [1,5] naphthyridin-10-yl 2-fluorobenzoate* (**7k**): Yield: 65%; pale yellow powder; m.p. 245.3–246.2 °C; IR, υ_max_ (KBr) cm^−1^: 1744 (C=O), 1675 (ester), 1420 (C=N). UV λ_max_ (lg ε) (DCM) 364 (4.36) nm, 382 (4.34) nm. ^1^H-NMR (500 MHz, CDCl_3_) δ 8.84 (d, *J* = 5.0 Hz, 1H), 8.72 (d, *J* = 8.8 Hz, 1H), 8.16 (td, *J* = 7.6, 1.9 Hz, 1H), 8.08–8.00 (m, 2H), 7.94 (d, *J* = 5.0 Hz, 1H), 7.68–7.62 (m, 1H), 7.57 (dd, *J* = 8.8, 2.3 Hz, 1H), 7.32 (td, *J* = 7.6, 1.1 Hz, 1H), 7.29–7.22 (m, 1H), 7.00 (d, *J* = 9.8 Hz, 1H). ^13^C-NMR (125 MHz, CDCl_3_) δ 162.9 (d, *J* = 4.0 Hz), 162.4 (d, *J* = 262.7 Hz), 159.4, 148.3, 146.0, 139.7, 137.1, 136.5, 135.6 (d, *J* = 262.7 Hz), 132.6, 129.8, 129.0, 125.5, 124.5, 124.3, 124.3, 118.0, 117.7 (d, *J* = 9.7 Hz), 117.4 (d, *J* = 22.4 Hz), 116.6, 116.2. ESI-MS *m*/*z*: [M + H]^+^_(found)_ = 359.34, [M + H]^+^_(calcd)_ = 359.33; Elemental analysis calculated for C_21_H_11_FN_2_O_3_ (%): C, 70.39; H, 3.09; N, 7.82 (found: C, 70.41; H, 3.06; N, 7.81).

*6-Oxo-6H-Indolo [3,2,1-de] [1,5] naphthyridin-10-yl 3-fluorobenzoate* (**7l**): Yield: 62%; pale yellow powder; m.p. 233.4–234.2 °C; IR, υ_max_ (KBr) cm^−1^: 1747 (C=O), 1675 (ester), 1422 (C=N). UV λ_max_ (lg ε) (DCM) 364 (4.16) nm, 382 (4.15) nm. ^1^H-NMR (500 MHz, CDCl_3_) δ 8.84 (d, *J* = 5.0 Hz, 1H), 8.73 (d, *J* = 8.8 Hz, 1H), 8.07–8.02 (m, 2H), 8.01 (d, *J* = 2.3 Hz, 1H), 7.95–7.90 (m, 2H), 7.57–7.51 (m, 2H), 7.41–7.36 (m, 1H), 7.00 (d, *J* = 9.8 Hz, 1H). ^13^C-NMR (125 MHz, CDCl_3_) δ 164.2 (d, *J* = 3.2 Hz), 163.7 (d, *J* = 247.0 Hz), 159.4, 148.4, 146.0, 139.7, 137.1, 136.6, 132.6, 131.3 (d, *J* = 7.7 Hz), 130.4 (d, *J* = 7.8 Hz), 129.7, 129.0, 126.0 (d, *J* = 3.1 Hz), 125.6, 124.4, 121.1 (d, *J* = 21.6 Hz), 118.1, 117.2 (d, *J* = 22.2 Hz), 116.6, 116.1. ESI-MS *m*/*z*: [M + H]^+^_(found)_ = 359.33, [M + H]^+^_(calcd)_ = 359.33; Elemental analysis calculated for C_21_H_11_FN_2_O_3_ (%): C, 70.39; H, 3.09; N, 7.82 (found: C, 70.44; H, 3.12; N, 7.79).

*6-Oxo-6H-Indolo [3,2,1-de] [1,5] naphthyridin-10-yl 4-fluorobenzoate* (**7m**): Yield: 66%; pale yellow powder; m.p. 226.2–227.4 °C; IR, υ_max_ (KBr) cm^−1^: 1736 (C=O), 1687 (ester), 1417 (C=N). UV λ_max_ (lg ε) (DCM) 364 (4.15) nm, 382 (4.12) nm. ^1^H-NMR (500 MHz, CDCl_3_) δ 8.83 (d, *J* = 5.0 Hz, 1H), 8.72 (d, *J* = 8.8 Hz, 1H), 8.30–8.24 (m, 2H), 8.03 (d, *J* = 9.8 Hz, 1H), 8.00 (d, *J* = 2.3 Hz, 1H), 7.92 (d, *J* = 5.0 Hz, 1H), 7.54 (dd, *J* = 8.8, 2.3 Hz, 1H), 7.25–7.20 (m, 2H), 6.99 (d, *J* = 9.8 Hz, 1H). ^13^C-NMR (125 MHz, CDCl_3_) δ 167.4 (d, *J* = 257.0 Hz), 164.3, 159.4, 148.5, 146.0, 139.7, 137.1, 136.5, 132.6, 132.4 (d, *J* = 9.6), 129.7, 129.0, 125.6, 125.4 (d, *J* = 3.0 Hz), 124.4, 118.0, 116.6, 116.2, 116.0 (d, *J* = 23.1 Hz). ESI-MS *m*/*z*: [M + H]^+^_(found)_ = 359.34, [M + H]^+^_(calcd)_ = 359.33; Elemental analysis calculated for C_21_H_11_FN_2_O_3_ (%): C, 70.39; H, 3.09; N, 7.82 (found: C, 70.26; H, 2.98; N, 7.87).

*6-Oxo-6H-Indolo [3,2,1-de] [1,5] naphthyridin-10-yl 2-(trifluoromethyl) benzoate* (**7n**): Yield: 61%; pale yellow powder; m.p. 191.3–192.1 °C; IR, υ_max_ (KBr) cm^−1^: 1754 (C=O), 1681 (ester), 1420 (C=N). UV λ_max_ (lg ε) (DCM) 363 (4.26) nm, 382 (4.24) nm. ^1^H-NMR (500 MHz, CDCl_3_) δ 8.85 (d, *J* = 5.0 Hz, 1H), 8.72 (d, *J* = 8.8 Hz, 1H), 8.08–8.00 (m, 3H), 7.97 (d, *J* = 5.0 Hz, 1H), 7.90–7.86 (m, 1H), 7.76–7.71 (m, 2H), 7.57 (dd, *J* = 8.8, 2.3 Hz, 1H), 6.99 (d, *J* = 9.8 Hz, 1H). ^13^C-NMR (125 MHz, CDCl_3_) δ 165.2, 159.4, 148.2, 146.0, 139.8, 137.2, 136.6, 132.6, 132.1, 132.0, 130.8, 130.0, 129.7, 129.2 (d, *J* = 42.5 Hz), 127.1 (d, *J* = 5.4 Hz), 127.1 (d, *J* = 5.5 Hz), 125.6, 124.1, 123.5 (d, *J* = 273.8 Hz), 118.1, 116.7, 115.8. ESI-MS *m*/*z*: [M + H]^+^_(found)_ = 409.38, [M + H]^+^_(calcd)_ = 409.34; Elemental analysis calculated for C_22_H_11_F_3_N_2_O_3_ (%): C, 64.71; H, 2.72; N, 6.86 (found: C, 64.66; H, 2.73; N, 6.89).

*6-Oxo-6H-Indolo [3,2,1-de] [1,5] naphthyridin-10-yl 3-(trifluoromethyl) benzoate* (**7o**): Yield: 62%; pale yellow powder; m.p. 199.9–200.8 °C; IR, υ_max_ (KBr) cm^−1^: 1742 (C=O), 1687 (ester), 1422 (C=N). UV λ_max_ (lg ε) (DCM) 364 (4.31) nm, 382 (4.33) nm. ^1^H-NMR (500 MHz, CDCl_3_) δ 8.84 (d, *J* = 5.0 Hz, 1H), 8.74 (d, *J* = 8.8 Hz, 1H), 8.52 (s, 1H), 8.44 (d, *J* = 7.8 Hz, 1H), 8.07–8.00 (m, 2H), 7.96–7.91 (m, 2H), 7.71 (t, *J* = 7.8 Hz, 1H), 7.57 (dd, *J* = 8.8, 2.3 Hz, 1H), 7.00 (d, *J* = 9.8 Hz, 1H). ^13^C-NMR (125 MHz, CDCl_3_) δ 164.0, 159.4, 148.3, 146.0, 139.8, 137.2, 136.6, 133.4, 132.6, 131.6 (d, *J* = 33.4 Hz), 130.4 (d, *J* = 3.8 Hz), 130.1, 129.6, 129.5, 129.0, 127.2 (d, *J* = 3.9 Hz), 125.6, 124.6 (d, *J* = 272.7 Hz), 124.3, 118.1, 116.6, 116.0. ESI-MS *m*/*z*: [M + H]^+^_(found)_ = 409.40, [M + H]^+^_(calcd)_ = 409.34; Elemental analysis calculated for C_22_H_11_F_3_N_2_O_3_ (%): C, 64.71; H, 2.72; N, 6.86 (found: C, 64.80; H, 2.74; N, 6.90).

*6-Oxo-6H-Indolo [3,2,1-de] [1,5] naphthyridin-10-yl 4-(trifluoromethyl) benzoate* (**7p**): Yield: 62%; pale yellow powder; m.p. 223.8–224.9 °C; IR, υ_max_ (KBr) cm^−1^: 1743 (C=O), 1677 (ester), 1415 (C=N). UV λ_max_ (lg ε) (DCM) 364 (4.21) nm, 382 (4.22) nm. ^1^H-NMR (500 MHz, CDCl_3_) δ 8.85 (d, *J* = 5.0 Hz, 1H), 8.74 (d, *J* = 8.8 Hz, 1H), 8.37 (d, *J* = 8.2 Hz, 2H), 8.07–7.99 (m, 2H), 7.94 (d, *J* = 5.0 Hz, 1H), 7.83 (d, *J* = 8.3 Hz, 2H), 7.56 (dd, *J* = 8.8, 2.3 Hz, 1H), 7.00 (d, *J* = 9.8 Hz, 1H). ^13^C-NMR (125 MHz, CDCl_3_) δ 164.1, 159.4, 148.3, 146.0, 139.8, 137.2, 136.6, 135.4 (d, *J* = 33.0 Hz), 132.6, 132.4, 130.7, 129.6, 129.0, 125.8 (d, *J* = 4.0 Hz), 125.6, 124.5 (d, *J* = 273.6 Hz), 124.3, 118.1, 116.6, 116.0. ESI-MS *m*/*z*: [M + H]^+^_(found)_ = 409.37, [M + H]^+^_(calcd)_ = 409.34; Elemental analysis calculated for C_22_H_11_F_3_N_2_O_3_ (%): C, 64.71; H, 2.72; N, 6.86 (found: C, 64.79; H, 2.77; N, 6.81).

*6-Oxo-6H-Indolo [3,2,1-de] [1,5] naphthyridin-10-yl 1-naphthoate* (**7q**): Yield: 83%; white powder; m.p. 221.3–222.4 °C; IR, υ_max_ (KBr) cm^−1^: 1732 (C=O), 1678 (ester), 1420 (C=N). UV λ_max_ (lg ε) (DCM) 364 (4.57) nm, 382 (4.55) nm. ^1^H-NMR (500 MHz, CDCl_3_) δ 9.06 (dd, *J* = 8.6, 1.1 Hz, 1H), 8.83 (d, *J* = 5.0 Hz, 1H), 8.74 (d, *J* = 8.7 Hz, 1H), 8.55 (dd, *J* = 7.3, 1.3 Hz, 1H), 8.14 (dt, *J* = 8.2, 1.1 Hz, 1H), 8.07 (d, *J* = 2.3 Hz, 1H), 8.03 (d, *J* = 9.8 Hz, 1H), 7.97–7.92 (m, 2H), 7.68–7.55 (m, 4H), 7.00 (d, *J* = 9.8 Hz, 1H). ^13^C-NMR (125 MHz, CDCl_3_) δ 165.8, 159.4, 148.7, 146.0, 139.7, 137.1, 136.5, 134.8, 134.0, 132.6, 131.8, 131.5, 129.8, 129.0, 128.8, 128.4, 126.6, 125.6, 125.6, 125.3, 124.7, 124.6, 118.1, 116.6, 116.4. ESI-MS *m*/*z*: [M + H]^+^_(found)_ = 391.39, [M + H]^+^_(calcd)_ = 391.40; Elemental analysis calculated for C_25_H_14_N_2_O_3_ (%): C, 76.92; H, 3.61; N, 7.18 (found: C, 76.99; H, 3.57; N, 7.24).

*6-Oxo-6H-Indolo [3,2,1-de] [1,5] naphthyridin-10-yl 2-naphthoate* (**7r**): Yield: 80%; white powder; m.p. 234.0–235.1 °C; IR, υ_max_ (KBr) cm^−1^: 1735 (C=O), 1685 (ester), 1419 (C=N). UV λ_max_ (lg ε) (DCM) 364 (4.59) nm, 382 (4.58) nm. ^1^H-NMR (500 MHz, CDCl_3_) δ 8.86–8.79 (m, 2H), 8.73 (d, *J* = 8.8 Hz, 1H), 8.22 (dd, *J* = 8.6, 1.7 Hz, 1H), 8.06 (d, *J* = 2.2 Hz, 1H), 8.03 (d, *J* = 9.8 Hz, 1H), 8.01 (d, *J* = 8.2, 1H), 7.96 (d, *J* = 8.6, 1H), 7.94–7.89 (m, 2H), 7.67–7.57 (m, 3H), 6.99 (d, *J* = 9.8 Hz, 1H). ^13^C-NMR (125 MHz, CDCl_3_) δ 165.4, 159.4, 148.8, 146.0, 139.7, 137.0, 136.5, 136.0, 132.6, 132.5, 132.2, 129.8, 129.5, 129.0, 128.9, 128.6, 127.9, 127.0, 126.3, 125.6, 125.4, 124.6, 118.0, 116.6, 116.3. ESI-MS *m*/*z*: [M + H]^+^_(found)_ = 391.38, [M + H]^+^_(calcd)_ = 391.40; Elemental analysis calculated for C_25_H_14_N_2_O_3_ (%): C, 76.92; H, 3.61; N, 7.18 (found: C, 76.85; H, 3.57; N, 7.12).

*6-Oxo-6H-Indolo [3,2,1-de] [1,5] naphthyridin-10-yl acetate* (**7s**): Yield: 52%; yellow solid; m.p. 198.5–199.3 °C; IR, υ_max_ (KBr) cm^−1^: 1758 (C=O), 1675 (ester), 1420 (C=N). UV λ_max_ (lg ε) (DCM) 364 (3.87) nm, 382 (3.85) nm. ^1^H-NMR (500 MHz, CDCl_3_) δ 8.80 (d, *J* = 5.0 Hz, 1H), 8.63 (d, *J* = 8.8 Hz, 1H), 8.00 (d, *J* = 9.8 Hz, 1H), 7.88 (d, *J* = 5.0 Hz, 1H), 7.85 (d, *J* = 2.2 Hz, 1H), 7.40 (dd, *J* = 8.8, 2.3 Hz, 1H), 6.96 (d, *J* = 9.8 Hz, 1H), 2.38 (s, 3H). ^13^C-NMR (125 MHz, CDCl_3_) δ 169.5, 159.3, 148.4, 145.9, 139.7, 136.9, 136.5, 132.5, 129.7, 129.0, 125.4, 124.3, 117.9, 116.6, 116.0, 21.2. ESI-MS *m*/*z*: [M + H]^+^_(found)_ = 279.26, [M + H]^+^_(calcd)_ = 279.27; Elemental analysis calculated for C_16_H_10_N_2_O_3_ (%): C, 69.06; H, 3.62; N, 10.07 (found: C, 69.01; H, 3.58; N, 10.13).

*6-Oxo-6H-Indolo [3,2,1-de] [1,5] naphthyridin-10-yl propionate* (**7t**): Yield: 51%; yellow solid; m.p. 166.7–168.1 °C; IR, υ_max_ (KBr) cm^−1^: 1753 (C=O), 1681 (ester), 1414 (C=N). UV λ_max_ (lg ε) (DCM) 364 (3.89) nm, 382 (3.85) nm. ^1^H-NMR (500 MHz, CDCl_3_) δ 8.81 (d, *J* = 5.0 Hz, 1H), 8.65 (d, *J* = 8.8 Hz, 1H), 8.01 (d, *J* = 9.8 Hz, 1H), 7.89 (d, *J* = 5.0 Hz, 1H), 7.86 (d, *J* = 2.2 Hz, 1H), 7.40 (dd, *J* = 8.8, 2.3 Hz, 1H), 6.97 (d, *J* = 9.8 Hz, 1H), 2.68 (q, *J* = 7.6 Hz, 2H), 1.32 (t, *J* = 7.6 Hz, 3H). ^13^C-NMR (125 MHz, CDCl_3_) δ 173.0, 159.3, 148.5, 145.9, 139.7, 136.9, 136.5, 132.5, 129.8, 129.0, 125.4, 124.4, 117.9, 116.6, 116.0, 27.8, 9.1. ESI-MS *m*/*z*: [M + H]^+^_(found)_ = 293.27, [M + H]^+^_(calcd)_ = 293.29; Elemental analysis calculated for C_17_H_12_N_2_O_3_ (%): C, 69.86; H, 4.14; N, 9.58 (found: C, 69.79; H, 4.17; N, 9.63).

*6-Oxo-6H-Indolo [3,2,1-de] [1,5] naphthyridin-10-yl butyrate* (**7u**): Yield: 55%; yellow solid; m.p. 138.2–139.5 °C; IR, υ_max_ (KBr) cm^−1^: 1753 (C=O), 1674 (ester), 1418 (C=N). UV λ_max_ (lg ε) (DCM) 364 (3.87) nm, 382 (3.86) nm. ^1^H-NMR (500 MHz, CDCl_3_) δ 8.83 (d, *J* = 5.0 Hz, 1H), 8.67 (d, *J* = 8.8 Hz, 1H), 8.02 (d, *J* = 9.8 Hz, 1H), 7.92 (d, *J* = 5.0 Hz, 1H), 7.87 (d, *J* = 2.2 Hz, 1H), 7.41 (dd, *J* = 8.8, 2.3 Hz, 1H), 6.98 (d, *J* = 9.8 Hz, 1H), 2.63 (t, *J* = 7.4 Hz, 2H), 1.89–1.81 (m, 2H), 1.10 (t, *J* = 7.4 Hz, 3H). ^13^C-NMR (125 MHz, CDCl_3_) δ 172.2, 159.4, 148.5, 145.9, 139.7, 136.9, 136.5, 132.6, 129.8, 129.0, 125.5, 124.4, 117.9, 116.6, 116.0, 36.3, 18.5, 13.7. ESI-MS *m*/*z*: [M + H]^+^_(found)_ = 307.28, [M + H]^+^_(calcd)_ = 307.32; Elemental analysis calculated for C_18_H_14_N_2_O_3_ (%): C, 70.58; H, 4.61; N, 9.15 (found: C, 70.55; H, 4.66; N, 9.18).

*6-Oxo-6H-Indolo [3,2,1-de] [1,5] naphthyridin-10-yl pentanoate* (**7v**): Yield: 56%; yellow solid; m.p. 141.6–142.7 °C; IR, υ_max_ (KBr) cm^−1^: 1755 (C=O), 1675 (ester), 1417 (C=N). UV λ_max_ (lg ε) (DCM) 364 (3.87) nm, 382 (3.85) nm. ^1^H-NMR (500 MHz, CDCl_3_) δ 8.81 (d, *J* = 5.0 Hz, 1H), 8.64 (d, *J* = 8.8 Hz, 1H), 8.00 (d, *J* = 9.8 Hz, 1H), 7.90 (d, *J* = 5.0 Hz, 1H), 7.84 (d, *J* = 2.3 Hz, 1H), 7.39 (dd, *J* = 8.8, 2.3 Hz, 1H), 6.96 (d, *J* = 9.8 Hz, 1H), 2.64 (t, *J* = 7.5 Hz, 2H), 1.83–1.76 (m, 2H), 1.54–1.45 (m, 2H), 1.01 (t, *J* = 7.4 Hz, 3H). ^13^C NMR (125 MHz, CDCl_3_) 172.4, 159.3, 148.5, 145.9, 139.6, 136.9, 136.5, 132.5, 129.7, 129.0, 125.4, 124.4, 117.9, 116.6, 116.0, 34.1, 27.0, 22.3, 13.8. ESI-MS *m*/*z*: [M + H]^+^_(found)_ = 321.29, [M + H]^+^_(calcd)_ = 321.35; Elemental analysis calculated for C_19_H_16_N_2_O_3_ (%): C, 71.24; H, 5.03; N, 8.74 (found: C, 71.16; H, 5.11; N, 8.69).

*6-Oxo-6H-Indolo [3,2,1-de] [1,5] naphthyridin-10-yl hexanoate* (**7w**): Yield: 53%; yellow solid; m.p. 112.8–113.7 °C; IR, υ_max_ (KBr) cm^−1^: 1761 (C=O), 1675 (ester), 1417 (C=N). UV λ_max_ (lg ε) (DCM) 364 (3.85) nm, 382 (3.86) nm. ^1^H-NMR (500 MHz, CDCl_3_) δ 8.81 (d, *J* = 5.0 Hz, 1H), 8.64 (d, *J* = 8.8 Hz, 1H), 8.01 (d, *J* = 9.8 Hz, 1H), 7.90 (d, *J* = 5.0 Hz, 1H), 7.85 (d, *J* = 2.3 Hz, 1H), 7.39 (dd, *J* = 8.8, 2.3 Hz, 1H), 6.97 (d, *J* = 9.8 Hz, 1H), 2.63 (t, *J* = 7.5 Hz, 2H), 1.85–1.78 (m, 2H), 1.48–1.38 (m, 4H), 0.96 (t, *J* = 7.1 Hz, 3H). ^13^C-NMR (126 MHz, CDCl_3_) δ 172.4, 159.3, 148.5, 145.9, 139.7, 136.9, 136.5, 132.5, 129.8, 129.0, 125.4, 124.4, 117.9, 116.6, 116.0, 34.4, 31.3, 24.6, 22.4, 13.9. ESI-MS *m*/*z*: [M + H]^+^_(found)_ = 335.35, [M + H]^+^_(calcd__)_ = 335.38; Elemental analysis calculated for C_20_H_18_N_2_O_3_ (%): C, 71.84; H, 5.43; N, 8.38 (found: C, 71.85; H, 5.46; N, 8.43).

*6-Oxo-6H-Indolo [3,2,1-de] [1,5] naphthyridin-10-yl dodecanoate* (**7x**): Yield: 49%; yellow solid; m.p. 65.4–66.6 °C; IR, υ_max_ (KBr) cm^−1^: 1757 (C=O), 1682 (ester), 1420 (C=N). UV λ_max_ (lg ε) (DCM) 364 (3.83) nm, 383 (3.82) nm. ^1^H-NMR (500 MHz, CDCl_3_) δ 8.84 (d, *J* = 5.0 Hz, 1H), 8.67 (d, *J* = 8.8 Hz, 1H), 8.07 (d, *J* = 9.8 Hz, 1H),7.94 (d, *J* = 5.0 Hz, 1H), 7.87 (d, *J* = 2.3 Hz, 1H), 7.41 (dd, *J* = 8.8, 2.3 Hz, 1H), 7.00 (d, *J* = 9.8 Hz, 1H), 2.64 (t, *J* = 7.5 Hz, 2H), 2.34 (t, *J* = 7.5 Hz, 4H), 1.85–1.76 (m, 2H), 1.69–1.57 (m, 4H), 1.53–1.41 (m, 2H), 0.90–0.86 (m, 9H). ^13^C-NMR (125 MHz, CDCl_3_) δ 172.4, 159.4, 148.5, 145.7, 139.5, 137.0, 136.4, 132.6, 130.0, 129.1, 125.4, 124.5, 118.0, 116.7, 116.1, 34.4, 34.1, 31.9, 29.6, 29.5, 29.3, 29.3, 29.1, 25.0, 24.8, 22.7. ESI-MS *m*/*z*: [M + H]^+^_(found)_ = 419.53, [M + H]^+^_(calcd)_ = 419.54; Elemental analysis calculated for C_26_H_30_N_2_O_3_ (%): C, 74.61; H, 7.23; N, 6.69 (found: C, 74.58; H, 7.19; N, 6.67).

### 3.3. Procedure for Determining Antifungal Activity

The *in vitro* antifungal activities of compounds **4**–**6** and **7a**–**7x** against three phytopathogenic fungi (*A. solani*, *F. graminearum*, and *F. solani*) were investigated using the mycelium linear growth rate method [24]. The test compounds and TBZ were completely dissolved in 0.5 mL of DMSO and the solution added to 9.5 mL of sterile water. The resulting solution was added to 90 mL of melted potato dextrose agar (PDA) medium at a temperature below 55 °C. After quickly and completely mixing, the medium containing the compounds at a concentration of 50 μg/mL was poured into a sterilized Petri dish for screening. DMSO without any compounds mixed with PDA served as the negative control. When the medium in the plates was partially solidified, a 4 mm thick, 5 mm diameter disc of fungus cut from previously subcultured Petri dishes was picked up and inoculated in the center of the PDA dishes. The dishes were kept in an incubator at 28 °C for 72 h. Three replicates were performed for each experiment. The inhibitory rate was calculated according to the following formula:

inhibition rate (%) = [(*d*_c_ − *d*_0_) − (*d*_s_ − *d*_0_)]/(*d*_c_ − *d*_0_) × 100% (1)

where *d*_0_ is the diameter of the fungus cut, *d*_c_ is the diameter of the untreated control fungus, and *d*_s_ is the diameter of the sample-treated fungus.

### 3.4. Procedure for Determining Antibacterial Activity

The test compounds were dissolved in dichloromethane at a concentration of 2 mg/mL, and then 5 μL of each solution was transferred onto sterile paper discs (6.0 mm diameter), so that the disc contained 10 μg of the compound. Melted LB agar medium (10 mL) was then mixed with 100 μL of bacterial suspension at 45 °C and poured into a sterilized Petri dish. The discs were placed gently on the surface of the media inoculated with the microorganism. Penicillin sodium was used as a positive control and dichloromethane as a negative control. The plates were allowed to stand for 2 h at 4 °C to allow for diffusion and then incubated at 37 °C for 12 h. The diameter of the zone of growth inhibition around the disc was measured. Experiments were repeated in triplicate.

The MICs of some synthesized compounds against four bacterial strains were evaluated using the micro-broth dilution method using Muller-Hinton broth in 96-well plates. The test compounds and penicillin sodium were first dissolved in DMSO at a concentration of 10 mg/mL, and then diluted 20-fold with sterile water to give the stock solution. An aliquot (50 μL) of this solution was pipetted into the first well of each line in a 96-well plate. The stock solution was then serially diluted to give two-fold serial dilutions of the test compounds and positive control in the subsequent wells. Then 50 μL of bacterial suspension (10^6^ cfu/mL) was added to each well and the plate set aside for incubation. One well containing bacterial cells and 2.5% DMSO was used as growth control, and another well containing only growth medium was used as a blank control. After incubation for 12 h at 37 °C, MICs were measured. Three replicates were performed for each experiment.

## 4. Conclusions

In this study, two naturally occurring canthine-6-ones and 24 novel ester derivatives of 10-hydroxycanthin-6-one were synthesized and their *in vitro* antimicrobial activity was evaluated. Most of the synthesized compounds showed potential antibacterial and antifungal activity. Compound **7s** represents a promising lead compound for further study, as it had significant antimicrobial activity against all the fungi and bacterial strains tested. Further research is required into more extensive structural modification, the structure-activity relationship and the mechanism of action of canthin-6-ones.

## Figures and Tables

**Table:** 

**7a**: R = C_6_H_5_	**7g**: R = 3′-ClC_6_H_4_	**7m**: R = 4′-FC_6_H_4_	**7s**: R = CH_3_
**7b**: R = 2′-pyridyl	**7h**: R = 4′-ClC_6_H_4_	**7n**: R = 2′-CF_3_C_6_H_4_	**7t**: R = CH_3_CH_2_
**7c**: R = 2′-CH_3_C_6_H_4_	**7i**: R = 2′,4′-Cl_2_C_6_H_3_	**70**: R = 3′-CF_3_C_6_H_4_	**7u**: R = CH_3_(CH_2_)_2_
**7d**: R = 3′-CH_3_C_6_H_4_	**7j**: R = 4′-CH_3_OC_6_H_4_	**7p**: R = 4′-CF_3_C_6_H_4_	**7v**: R = CH_3_(CH_2_)_3_
**7e**: R = 4′-CH_3_C_6_H_4_	**7k**: R = 2′-FC_6_H_4_	**7q**: R = 1′-naphthyl	**7w**: R = CH_3_(CH_2_)_4_
**7f**: R = 2′-ClC_6_H_4_	**7l**: R = 3′-FC_6_H_4_	**7r**: R = 2′-naphthyl	**7x**: R = CH_3_(CH_2_)_10_

**Table 1:** Average inhibition rates of compounds **4**–**6** and **7a**–**7x** against three phytopathogenic fungi.

Compounds		Average Inhibition Rate ± SD (%)		
No.	R	*A. solani*	*F. graminearum*	*F. solani*
**4**	-	61.2 ± 0.4 g	100.0 ± 0.0 a	53.4 ± 0.7 n
**5**	-	71.8 ± 0.5 b	77.7 ± 0.4 d	92.9 ± 0.1 a
**6**	-	2.0 ± 0.5 q	74.5 ± 0.5 e	57.9 ± 0.6 k
**7a**	C_6_H_5_	53.1 ± 0.7 i	48.8 ± 0.4 j	41.7 ± 0.7 q
**7b**	2′-pyridyl	66.6 ± 0.0 de	47.0 ± 0.7 k	79.8 ± 0.5 d
**7c**	2′-CH_3_C_6_H_4_	2.7 ± 0.7 q	35.6 ± 0.4 n	65.3 ± 1.5 i
**7d**	3′-CH_3_C_6_H_4_	45.8 ± 0.9 k	43.6 ± 0.6 l	64.1 ± 0.4 j
**7e**	4′-CH_3_C_6_H_4_	28.7 ± 0.8 l	23.6 ± 0.2 q	77.0 ± 0.6 e
**7f**	2′-ClC_6_H_4_	2.1 ± 0.8 q	31.8 ± 0.4 p	68.1 ± 0.4 h
**7g**	3′-ClC_6_H_4_	21.4 ± 0.8 n	3.4 ± 0.3 s	86.1 ± 0.6 c
**7h**	4′-ClC_6_H_4_	5.7 ± 0.9 p	3.1 ± 0.6 s	72.2 ± 0.5 g
**7i**	2′,4′-Cl_2_C_6_H_3_	50.1 ± 0.8 j	7.1 ± 0.5 r	22.8 ± 0.6 u
**7j**	4′-CH_3_OC_6_H_4_	50.6 ± 0.3 j	6.3 ± 0.4 r	41.9 ± 0.3 q
**7k**	2′-FC_6_H_4_	68.4 ± 0.7 c	37.6 ± 0.5 m	40.5 ± 0.4 r
**7l**	3′-FC_6_H_4_	54.0 ± 0.9 i	33.0 ± 0.7 o	49.4 ± 0.2 p
**7m**	4′-FC_6_H_4_	58.9 ± 1.3 h	51.1 ± 0.6 h	52.1 ± 0.9 o
**7n**	2′-CF_3_C_6_H_4_	64.4 ± 0.9 f	80.5 ± 0.4 c	13.5 ± 1.0 w
**7o**	3′-CF_3_C_6_H_4_	25.7 ± 0.7 m	32.6 ± 0.8 po	54.6 ± 0.3 m
**7p**	4′-CF_3_C_6_H_4_	8.3 ± 0.3 o	80.1 ± 1.0 c	22.9 ± 0.4 u
**7q**	1′-naphthyl	65.1 ± 1.1 ef	49.9 ± 0.6 i	25.2 ± 0.7 t
**7r**	2′-naphthyl	25.8 ± 0.2 m	50.2 ± 0.6 hi	20.3 ± 0.1 v
**7s**	CH_3_	68.4 ± 0.6 c	100.0 ± 0.0 a	86.0 ± 0.2 c
**7t**	CH_3_CH_2_	59.1 ± 0.9 h	70.0 ± 0.3 f	80.1 ± 0.3 d
**7u**	CH_3_(CH_2_)_2_	66.9 ± 0.9 cd	44.4 ± 0.7 l	69.2 ± 0.9 h
**7v**	CH_3_(CH_2_)_3_	49.9 ± 0.8 j	85.4 ± 0.4 b	74.2 ± 0.2 f
**7w**	CH_3_(CH_2_)_4_	66.5 ± 0.9 de	55.1 ± 0.2 g	56.6 ± 0.3 l
**7x**	CH_3_(CH_2_)_10_	72.2 ± 0.2 b	85.6 ± 0.4 b	29.8 ± 0.4 s
TBZ	-	89.1 ± 0.6 a	100.0 ± 0.0 a	89.3 ± 0.4 b

All values are expressed as means ± SD (*n* = 3). Tested fungi were treated with each compound at a concentration of 50 μg/mL for 72 h. The differences between data with the different lower case letters within a column are significant for the same tested fungus (*p* < 0.05).

**Table 2:** Inhibition zones (mm) of some synthesized compounds (10 μg).

Compounds	Gram-Positive Bacteria		Gram-Negative Bacteria	
	*B. cereus*	*B. subtilis*	*R. solanacearum*	*P. syringae*
**5**	11.2 ± 0.5	10.9 ± 0.8	12.6 ± 0.4	10.9 ± 1.0
**6**	- *	9.8 ± 0.4	-	8.5 ± 0.1
**7s**	12.2 ± 0.1	16.9 ± 0.7	12.3 ± 0.2	18.4 ± 0.2
**7t**	16.6 ± 0.2	17.4 ± 0.1	13.0 ± 0.2	21.0 ± 0.4
**7u**	13.5 ± 0.3	13.9 ± 0.4	-	13.0 ± 0.8
**7v**	12.7 ± 0.2	10.3 ± 1.1	-	15.4 ± 0.2
**7w**	9.4 ± 0.1	8.5 ± 0.3	-	12.5 ± 0.2
**Penicillin Sodium**	-	22.5 ± 0.2	16.2 ± 0.2	20.4 ± 0.8

All values are expressed as means ± SD (*n* = 3). * Inhibition zone < 8 mm.

**Table 3:** The *in vitro* antibacterial activity as MIC (μg/mL) for compounds **5**, **6**, and **7s**–**7w**.

Compounds	Gram-Positive Bacteria		Gram-Negative Bacteria	
	*B. cereus*	*B. subtilis*	*R. solanacearum*	*P. syringae*
**5**	3.91	7.81	15.62	7.81
**6**	15.62	>125	>125	>125
**7s**	15.62	15.62	31.25	7.81
**7t**	15.62	7.81	15.62	7.81
**7u**	31.25	31.25	ND *	15.62
**7v**	>125	125	ND	31.25
**7w**	>125	62.5	ND	>125
**Penicillin Sodium**	3.91	7.81	7.81	0.98

* Not determined.

## Supplementary Materials

Supplementary materials can be accessed at: http://www.mdpi.com/1420-3049/21/3/390/s1.
