# Supplementary material for: Synthesis and Evaluation of Ester Derivatives of 10-Hydroxycanthin-6-one as Potential Antimicrobial Agents
Source: Molecules. 2016 Mar 21;21(3):390. doi: 10.3390/molecules21030390 (PMC6272828; doi:10.3390/molecules21030390)
Supplement: Supplementary file 1 [file molecules-21-00390-s001.pdf]

Supplementary Materials: Synthesis and Evaluation of Ester Derivatives of 10-Hydroxycanthin-6-one as Potential Antimicrobial Agents

Fei Zhao, Jiang-Kun Dai, Dan Liu, Shi-Jun Wang, and Jun-Ru Wang

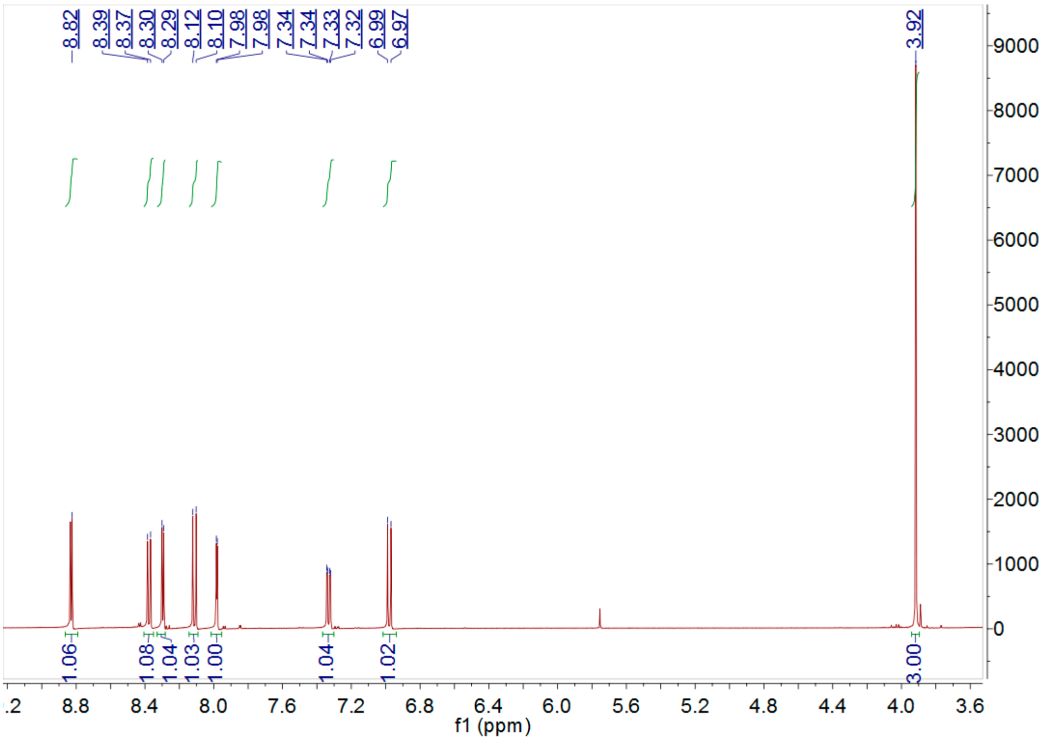

Figure S1. ^{1}H-NMR spectrum of compound **5**.

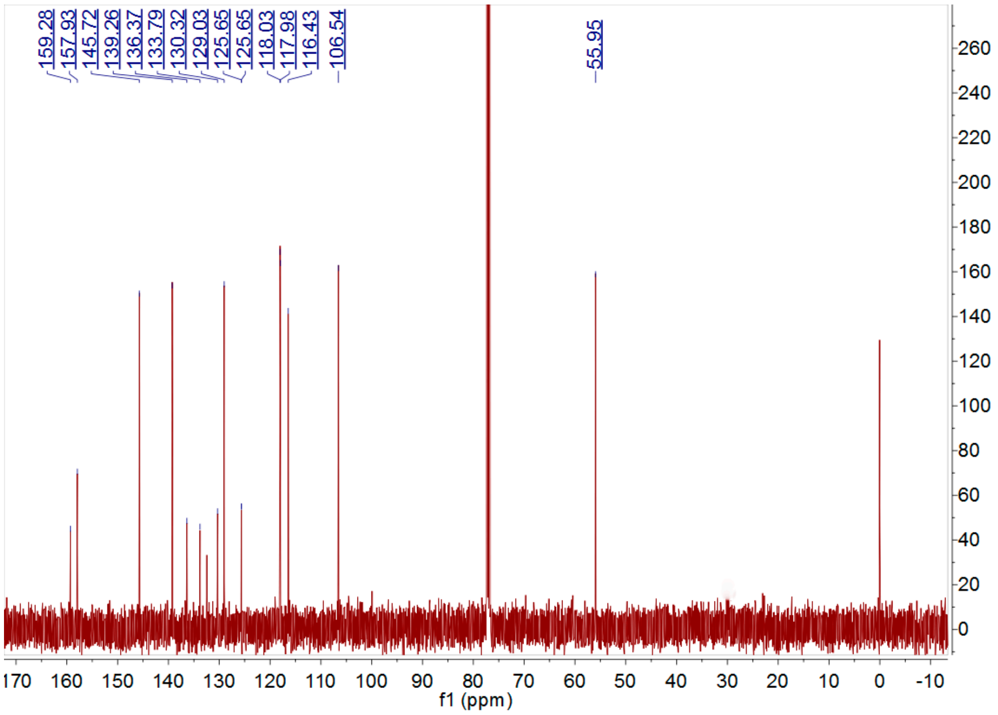

Figure S2. ^{13}C-NMR spectrum of compound **5**.

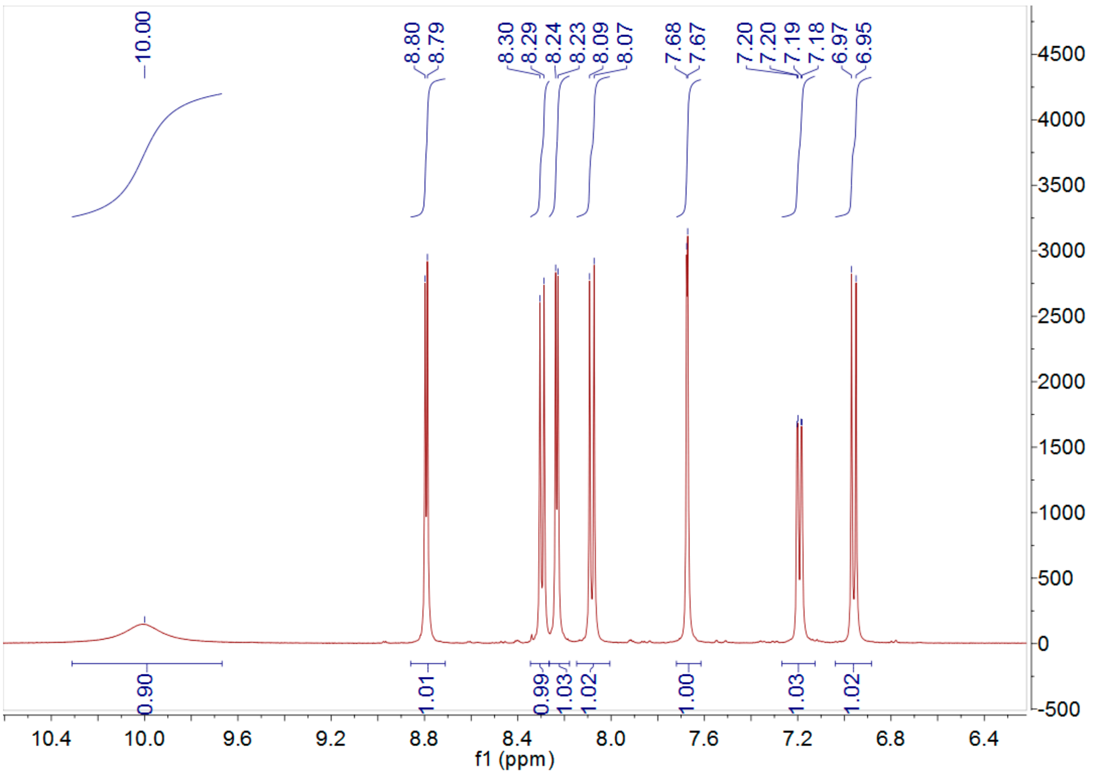

Figure S3. ^{1}H-NMR spectrum of compound **6**.

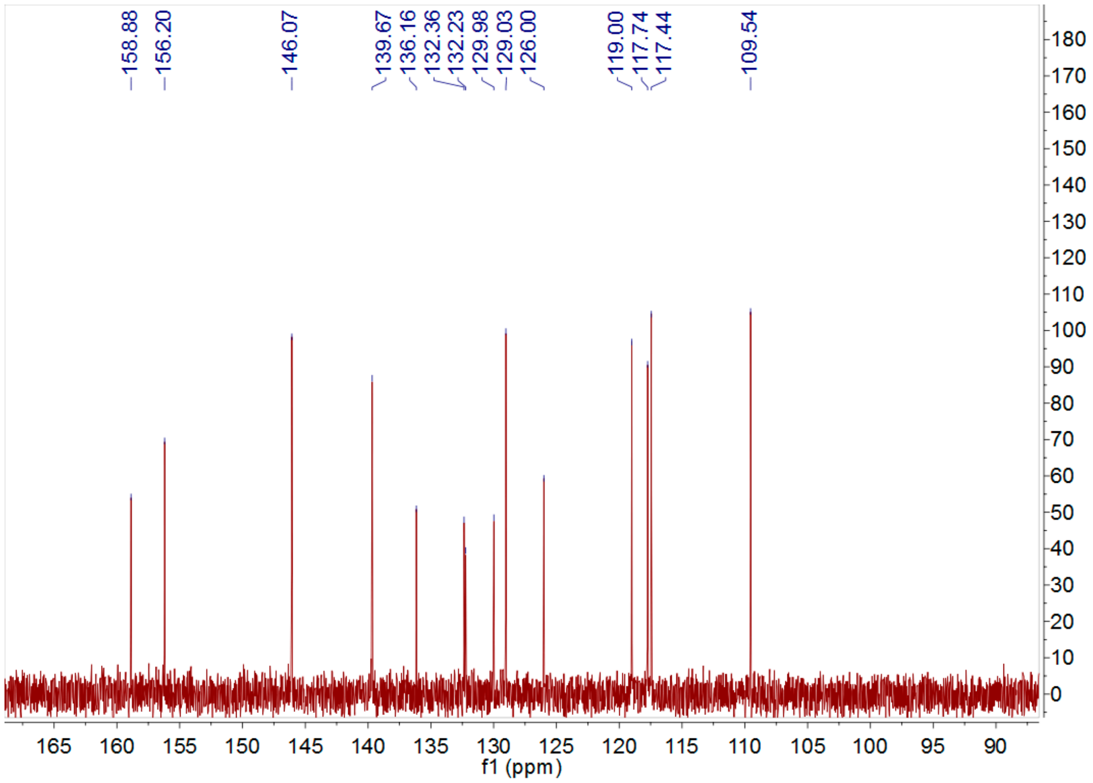

Figure S4. ^{13}C-NMR spectrum of compound **6**.

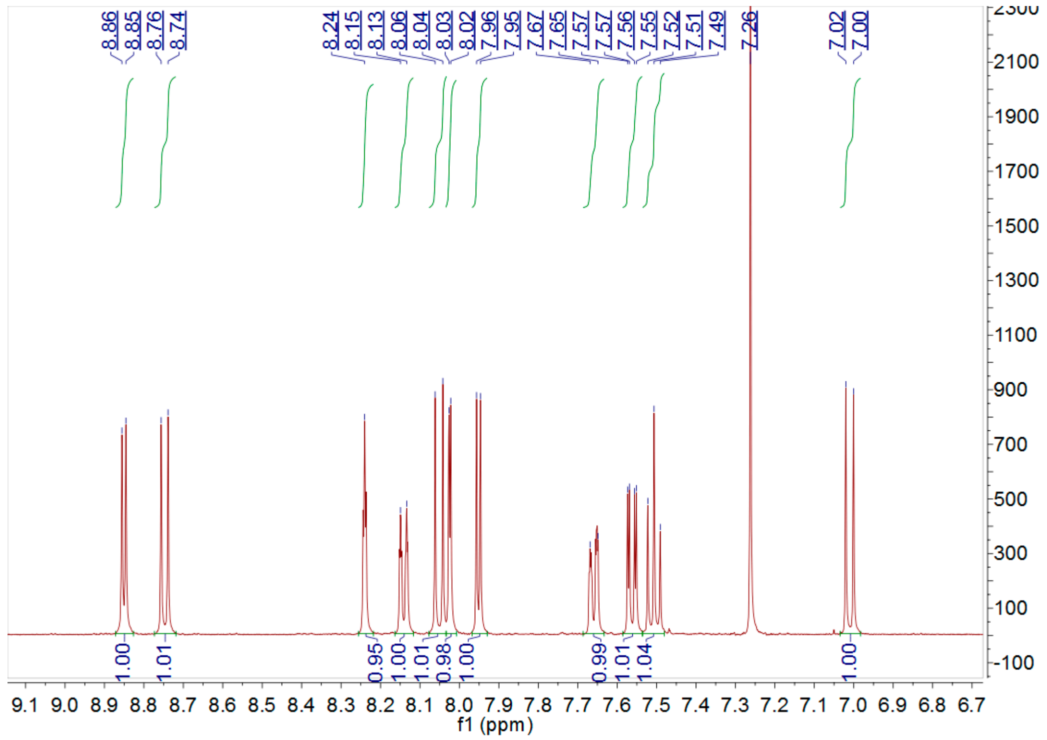

Figure S5. ^{1}H-NMR spectrum of compound **7g**.

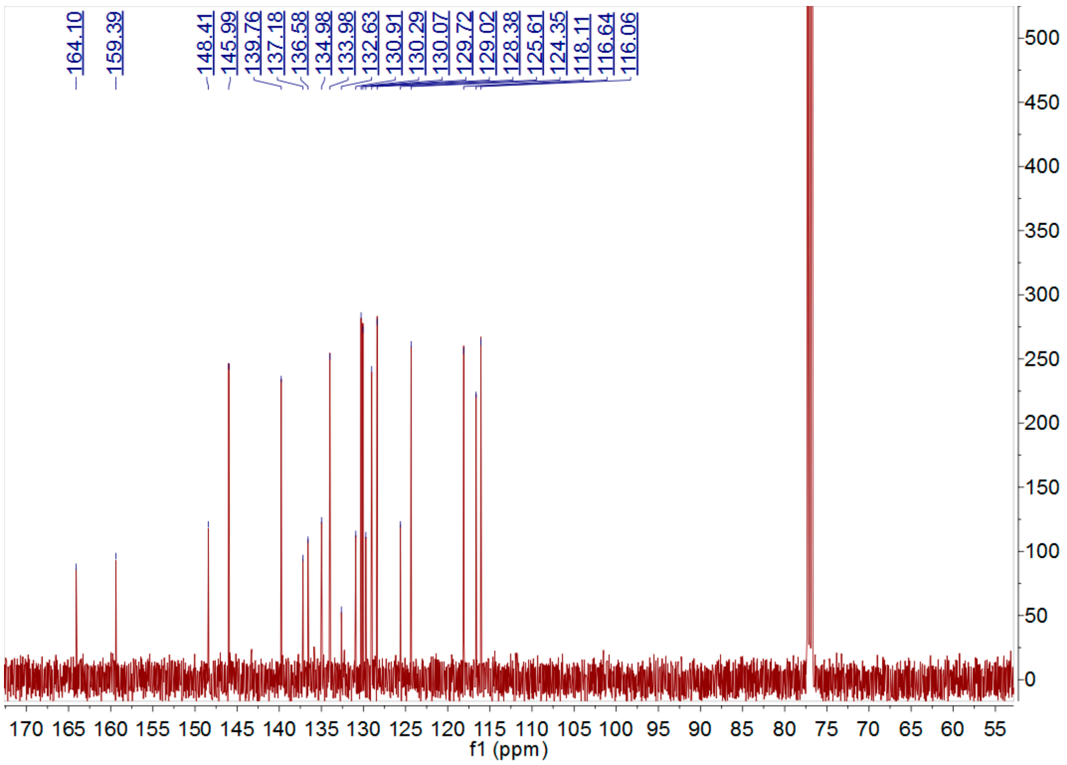

Figure S6. ^{13}C-NMR spectrum of compound **7g**.

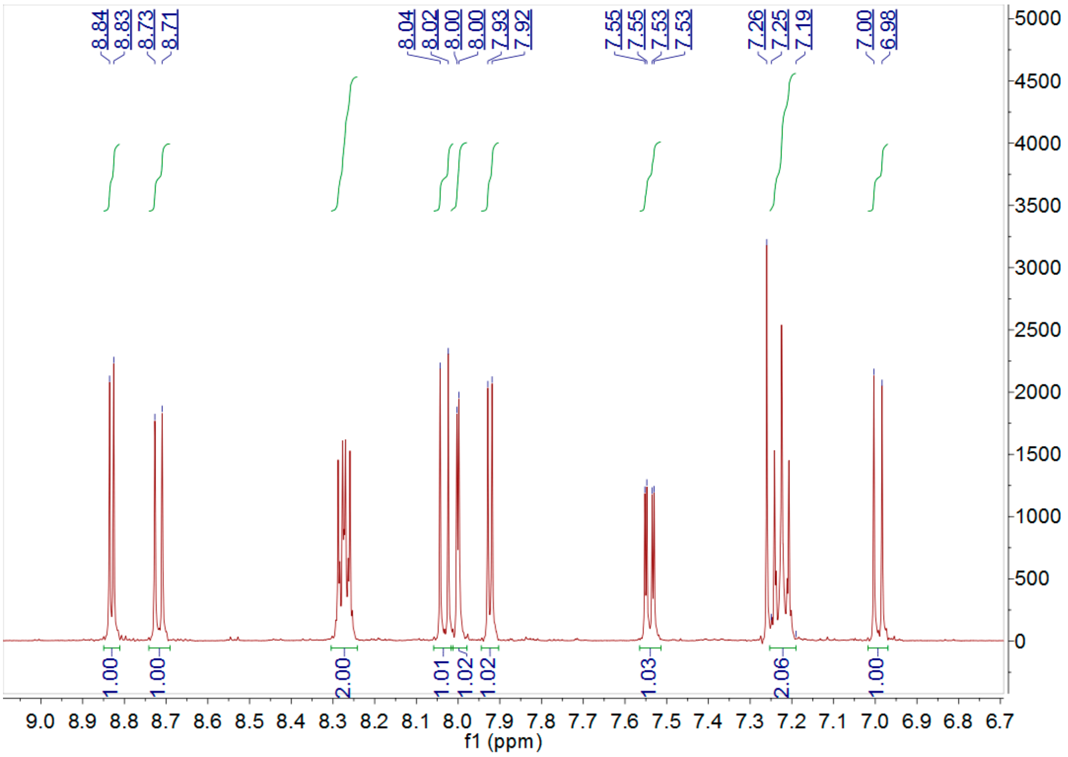

Figure S7. ^{1}H-NMR spectrum of compound **7m**.

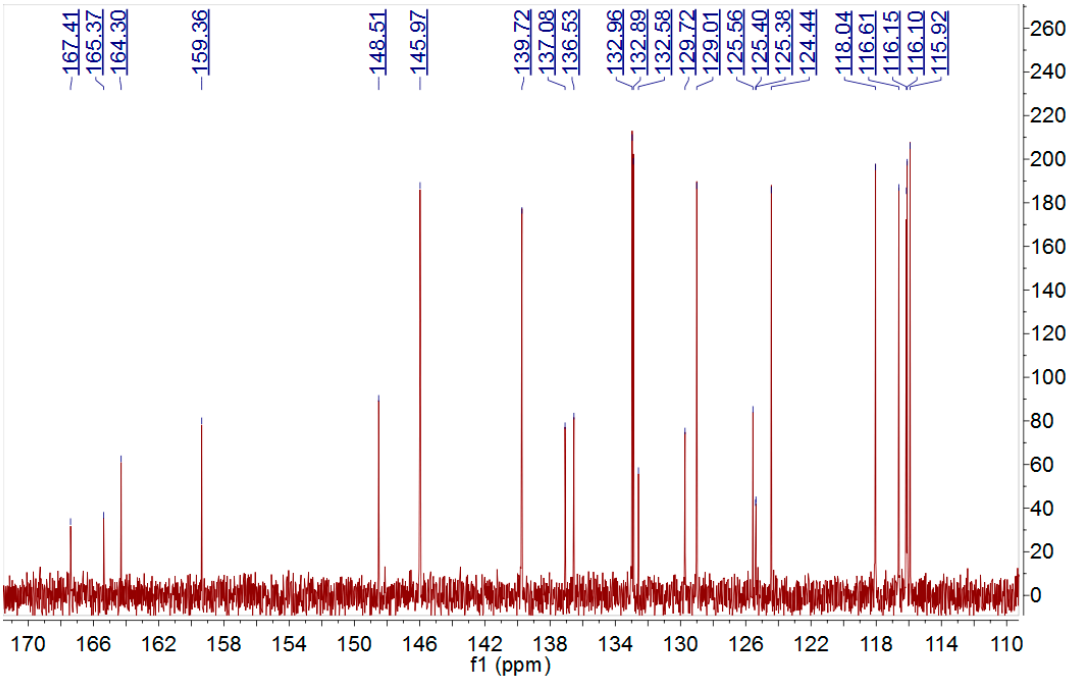

Figure S8. ^{13}C-NMR spectrum of compound **7m**.

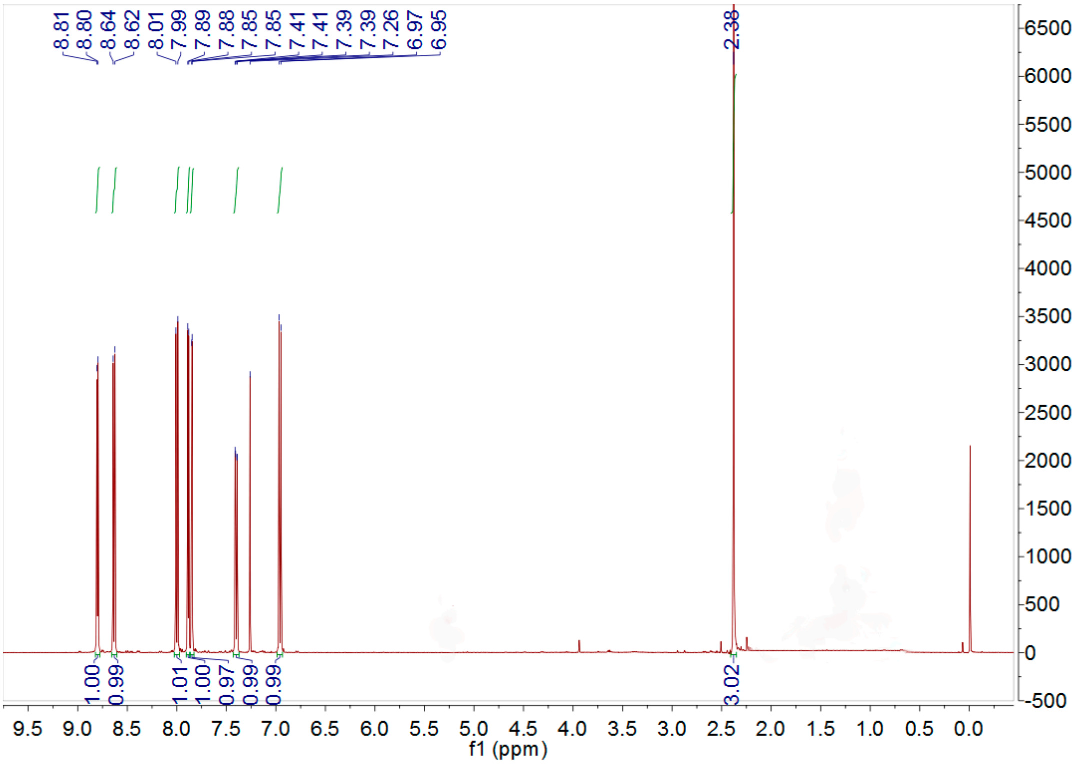

Figure S9. ^{1}H-NMR spectrum of compound **7s**.

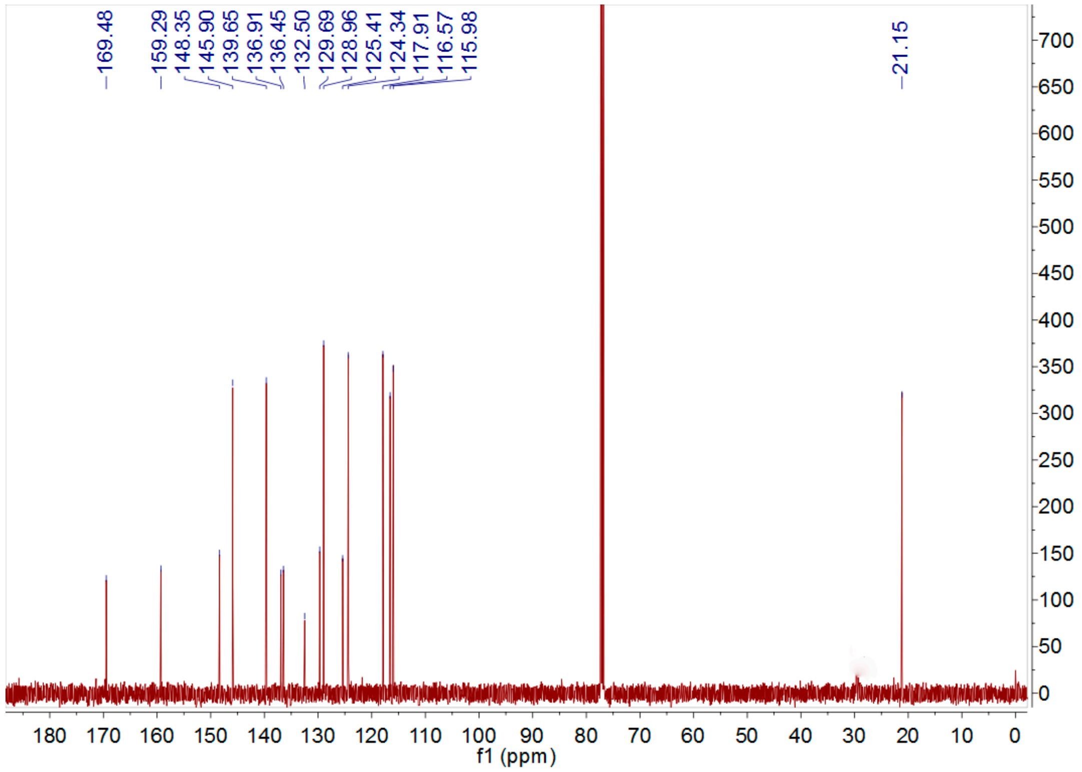

Figure S10. ^{13}C-NMR spectrum of compound **7s**.

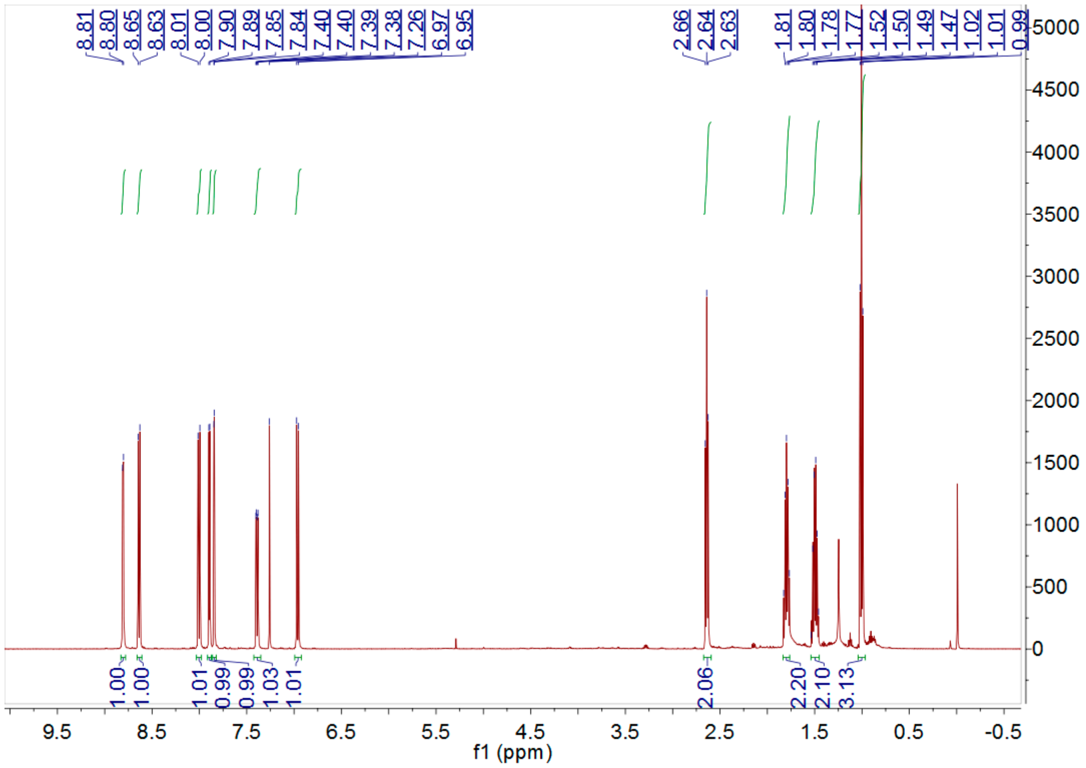

Figure S11. ^{1}H-NMR spectrum of compound **7v**.

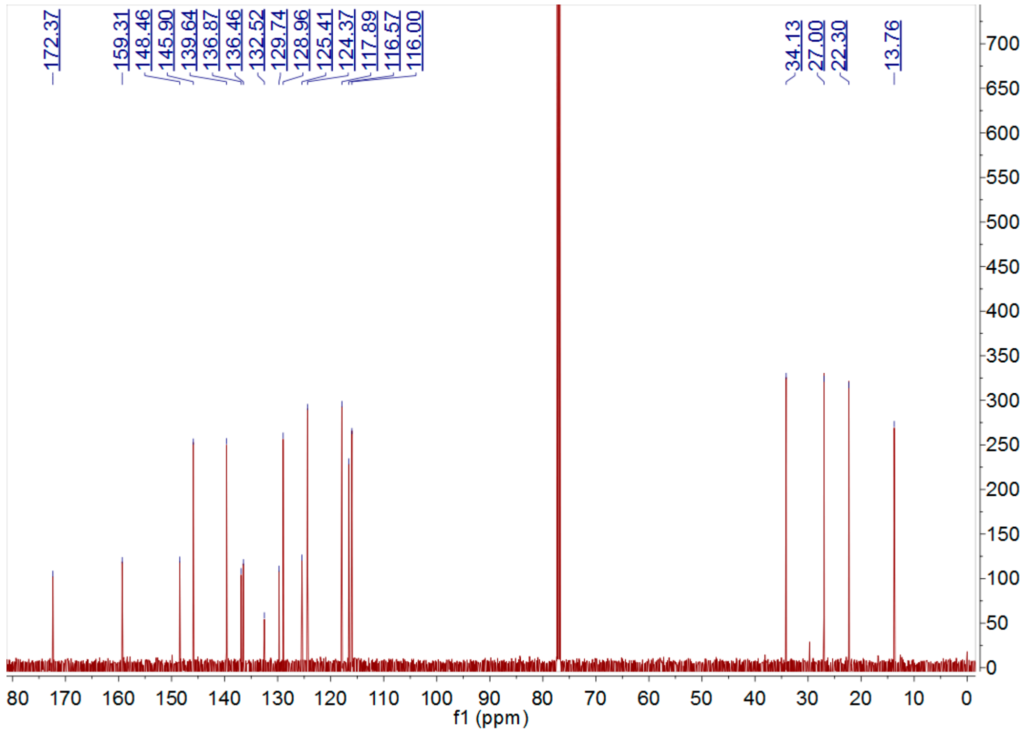

Figure S12. ^{13}C-NMR spectrum of compound **7v**.

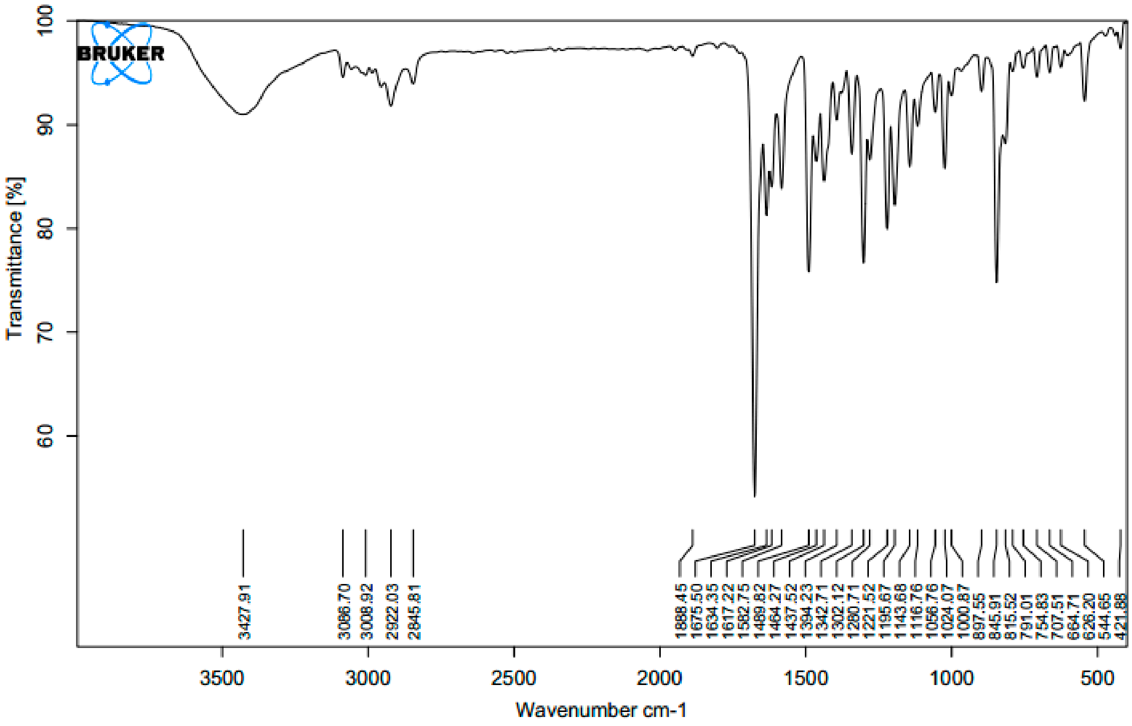

Figure S13. FT-IR spectrum of compound **5**.

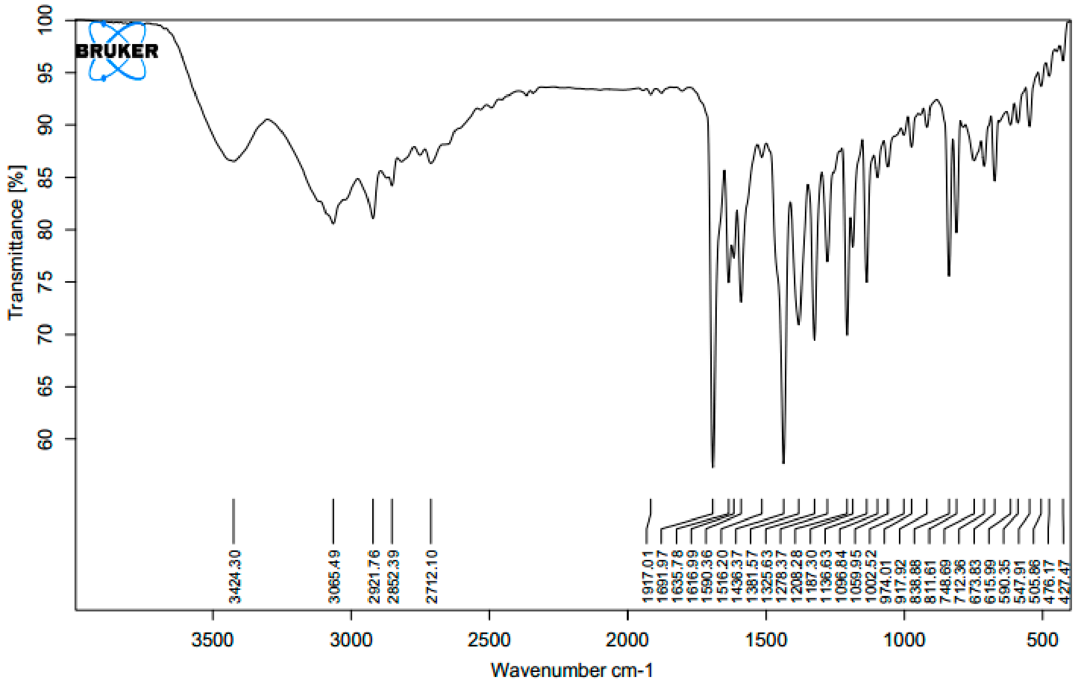

Figure S14. FT-IR spectrum of compound **6**.

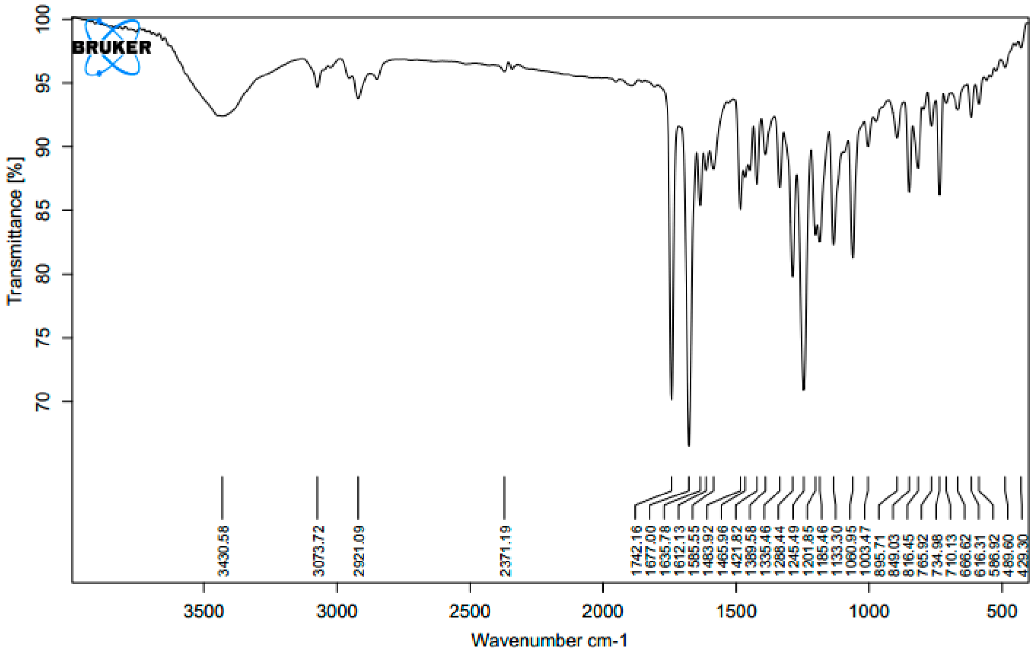

Figure S15. FT-IR spectrum of compound **7g**.

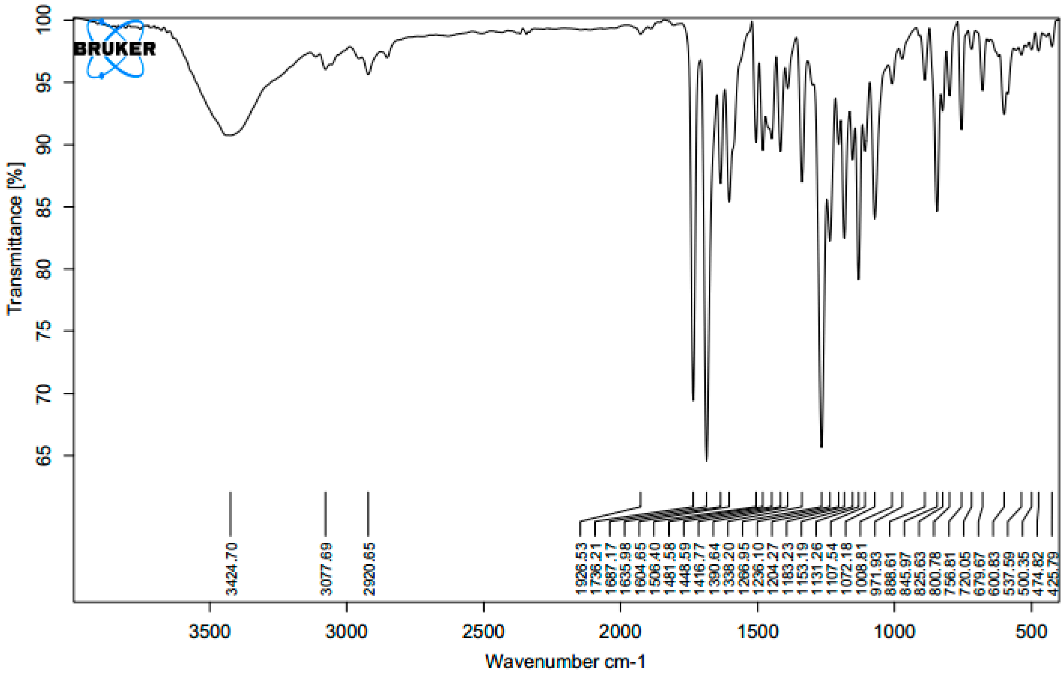

Figure S16. FT-IR spectrum of compound **7m**.

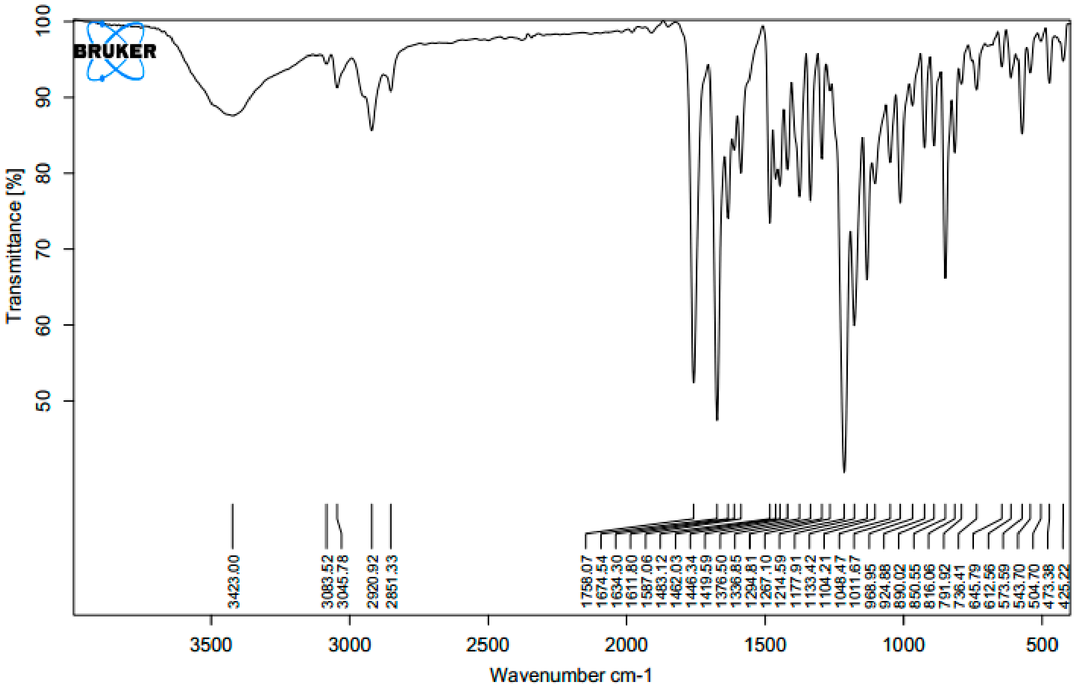

Figure S17. FT-IR spectrum of compound **7s**.

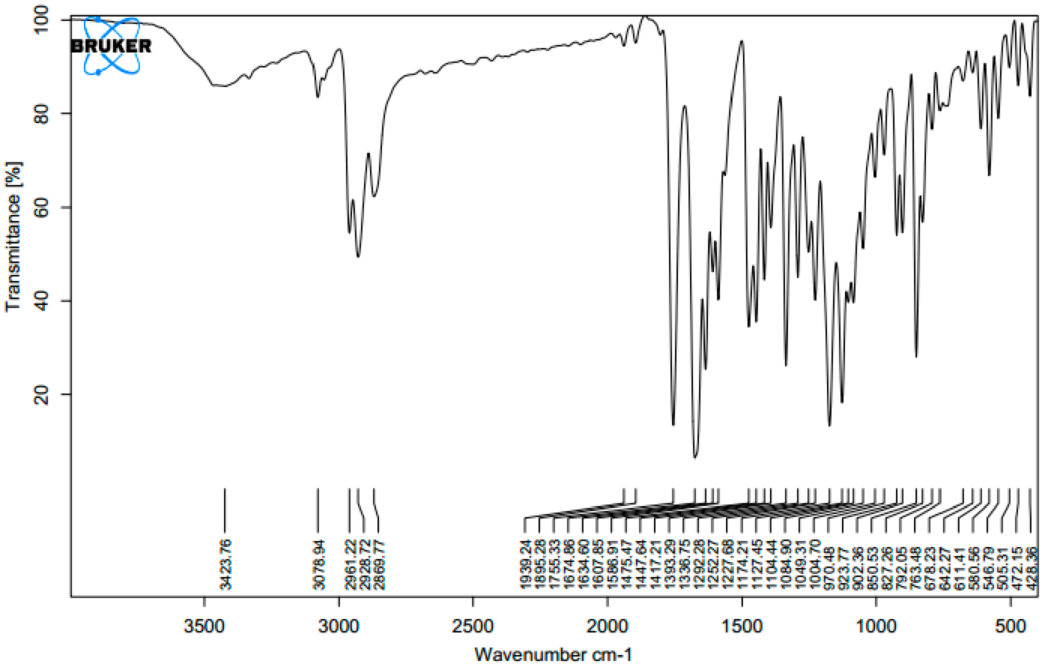

Figure S18. FT-IR spectrum of compound **7v**.
